# Early prediction of hypertensive disorders of pregnancy toward preventive early intervention

**DOI:** 10.1016/j.xagr.2024.100383

**Published:** 2024-07-27

**Authors:** Satoshi Mizuno, Satoshi Nagaie, Junichi Sugawara, Gen Tamiya, Taku Obara, Mami Ishikuro, Shinichi Kuriyama, Nobuo Yaegashi, Hiroshi Tanaka, Masayuki Yamamoto, Soichi Ogishima

**Affiliations:** 1 Department of Informatics for Genomic Medicine, Tohoku Medical Megabank Organization, Tohoku University, Miyagi, Japan (Mizuno, Nagaie, and Ogishima); 2 Department of Feto-Maternal Medical Science, Tohoku Medical Megabank Organization, Tohoku University. Department of Gynecology and Obstetrics, Tohoku University Graduate School of Medicine, Tohoku University, Miyagi, Japan (Sugawara); 3 Department of Statistical Genetics and Genomics, Tohoku Medical Megabank Organization, Tohoku University, Miyagi, Japan (Tamiya); 4 Department of Molecular Epidemiology, Tohoku Medical Megabank Organization, Tohoku University, Miyagi, Japan (Obara, Ishikuro, and Kuriyama); 5 Department of Gynecology and Obstetrics, Tohoku University Graduate School of Medicine, Tohoku University, Miyagi, Japan (Yaegashi); 6 Department of Bioclinical Informatics, Tohoku Medical Megabank Organization, Tohoku University, Miyagi, Japan (Tanaka); 7 Department of Integrative Genomics, Tohoku Medical Megabank Organization and Department of Medical Biochemistry, Graduate School of Medicine, Tohoku University, Miyagi, Japan (Yamamoto)

**Keywords:** hypertensive disorders of pregnancy, disease prediction, lifestyle, machine learning, obstetrics

## Abstract

**Background:**

Various disease prediction models have been developed, capitalizing on the wide use of electronic health records, but environmental factors that are important in the development of noncommunicable diseases are rarely included in the prediction models. Hypertensive disorders of pregnancy are leading causes of maternal morbidity and mortality and are known to cause several serious complications later in life.

**Objective:**

This study aims to develop early hypertensive disorders of pregnancy prediction models using comprehensive environmental factors based on self-report questionnaires in early pregnancy.

**Study Design:**

We developed machine learning and artificial intelligence models for the early prediction of hypertensive disorders of pregnancy using early pregnancy data from approximately 23,000 pregnancies in the Tohoku Medical Megabank Birth and Three Generation Cohort Study. We clarified the important features for prediction based on regression coefficients or Gini coefficients of the interpretable artificial intelligence models (i.e., logistic regression, random forest and XGBoost models) among our developed models.

**Results:**

The performance of the early hypertensive disorders of pregnancy prediction models reached an area under the receiver operating characteristic curve of 0.93, demonstrating that the early hypertensive disorders of pregnancy prediction models developed in this study retain sufficient performance in hypertensive disorders of pregnancy prediction. Among the early prediction models, the best performing model was based on self-reported questionnaire data in early pregnancy (mean of 20.2 gestational weeks at filling) which consist of comprehensive lifestyles. The interpretation of the models reveals that both eating habits were dominantly important for prediction.

**Conclusion:**

We have developed high-performance models for early hypertensive disorders of pregnancy prediction using large-scale cohort data from the Tohoku Medical Megabank project. Our study clearly revealed that the use of comprehensive lifestyles from self-report questionnaires led us to predict hypertensive disorders of pregnancy risk at the early stages of pregnancy, which will aid early intervention to reduce the risk of hypertensive disorders of pregnancy.

**AJOG Global Reports at a Glance**

**Why was this study conducted?**

Early prediction models of hypertensive disorders of pregnancy (HDP) for self-estimation of the risk have not been established but are indispensable to reduce the development of HDP and adverse outcomes.

**Key findings**

The key finding of our study is that we achieved development of high-performance artificial-intelligence (AI) models to predict HDP based on comprehensive environmental factors. The interpretation of our models clarifies the important lifestyle factors related to the onset and severity of HDP.

**What does this add to what is known?**

The novel points of our developed models are that the higher performances with AUCs of up to 0.93 based on a larger population size of comprehensive lifestyles than models in the past studies. Based on the above points, our developed AI models demonstrate the high possibility of the prediction of HDP in the early stage of pregnancy.

## Introduction

One of the most important aims in precision medicine is to develop quantitative models that ensure an accurate and elaborate evaluation of disease risks to predict and potentially prevent diseases in patients by using the models.^1^ More recently, many studies on machine learning and AI models of disease prediction have been performed utilizing large-scale data from electronic health records (EHRs),^2^ especially using subjects with target phenotypic abnormalities identified by phenotyping algorithms^3^ as training labels.

The Birth and Three-Generation Cohort Study (BirThree Cohort Study) of the Tohoku Medical Megabank Project (TMM)^4^ is a large-scale prospective cohort study that has recruited more than 73,000 participants, including more than 22,000 pregnant women and their children, partners and other family members. The TMM BirThree Cohort Study is an excellent data source for developing a disease prediction model because the integrated database of its study includes various kinds of data, such as laboratory tests, clinical records and environmental factors, including lifestyles and eating habits, that are related to disease risk in many noncommunicable diseases.^5^

Hypertensive disorders of pregnancy (HDP) are one of the major complications during pregnancy, affecting approximately 2%−8% of all pregnant patients,^6^ and are known to cause several serious complications later in life.^7^ According to the American College of Obstetricians and Gynecologists (ACOG) mainly classifies HDP into three subtypes: gestational hypertension (GH), preeclampsia (PE), and superimposed PE (SPE).^8^ One major difference between the subtypes is the occurrence of severe complications. Patients with PE and SPE are more likely to develop severe complications, including premature abruption and HELLP syndrome, which are leading causes of maternal and child morbidity and mortality.^9^ HDP is known as a multifactorial disease in which both environmental factors, specifically lifestyles, and genetic factors play important roles in the development of disease.^10^^,^^11^

The early prediction of PE among the subtypes of HDP has been addressed,^12^^,^^13^ and many early PE prediction models have been developed using omics data, biomarkers,^14^ combinations of clinical data^13^ and combinations of both clinical and laboratory data.^15^ However, a precise prediction model that can be applied for clinical use has not been established,^16^ and we surmise that there are serious limitations, such as sample size in up to a few dozen to hundred subjects and models in using linear models, which are known to be inferior to nonlinear models for multivariate distribution.^17^ Additionally, no or only a few environmental factors known to be associated with the risk of many multifactorial diseases were used to develop the models in previous studies. Other subtypes than PE are also known to have an important impact on obstetric outcomes.^18^ In particular, early detection of SPE is important for clinical practice because of the requirement of additional management of chronic hypertension to PE conditions for SPE subjects. Thus, we decided to develop early prediction models of HDP as well as its subtypes with laboratory test and environmental factor data collected in the early stage of pregnancy from approximately 22,000 pregnant women of the TMM BirThree Cohort Study. We also interpret the developed models to clarify the important factors for prediction.

## Material and methods

### Data sources

We selected research subjects from 23,790 pregnant women who were included in the TMM BirThree Cohort Study. We used laboratory test data collected in the early stage of pregnancy (mean of 21.09 gestational weeks (6.6 SD)) and questionnaires answered in the early stage of pregnancy (mean of 20.2 gestational weeks (7.9 SD), which are much earlier than the mean gestational weeks of HDP onset (33.3 (6.0 SD) gestational weeks) (Supplementary Figure 1). The details of the data collection and collected items were described in the previous report of the TMM BirThree Cohort Study.^19^

From 23,790 subjects, we excluded subjects who did not have medical records at delivery (n = 1,009), did not have a live birth (n = 167), withdrew from the study (n = 80), did not have blood pressure and proteinuria (PU) data (n = 82), and subjects having chronic hypertension (n = 542). Using these criteria, we included 21,910 research subjects in our dataset. We excluded subjects having chronic hypertension for the following reasons: 1) the inclusion of chronic hypertension in HDP is controversial because of the lack of international consensus, and 2) the prediction of chronic hypertension is useless because it can be identified before conception.

### Phenotyping of subjects

To identify HDP subtypes, we conducted phenotyping by using our rule-based phenotyping algorithm based on the diagnostic guidelines of the American College of Obstetricians and Gynecologists (ACOG)^8^ (see the details in the “Details of the rules for identifying HDP subtypes” section in the supplementary methods).

### Development of prediction models

**Figure 1:**
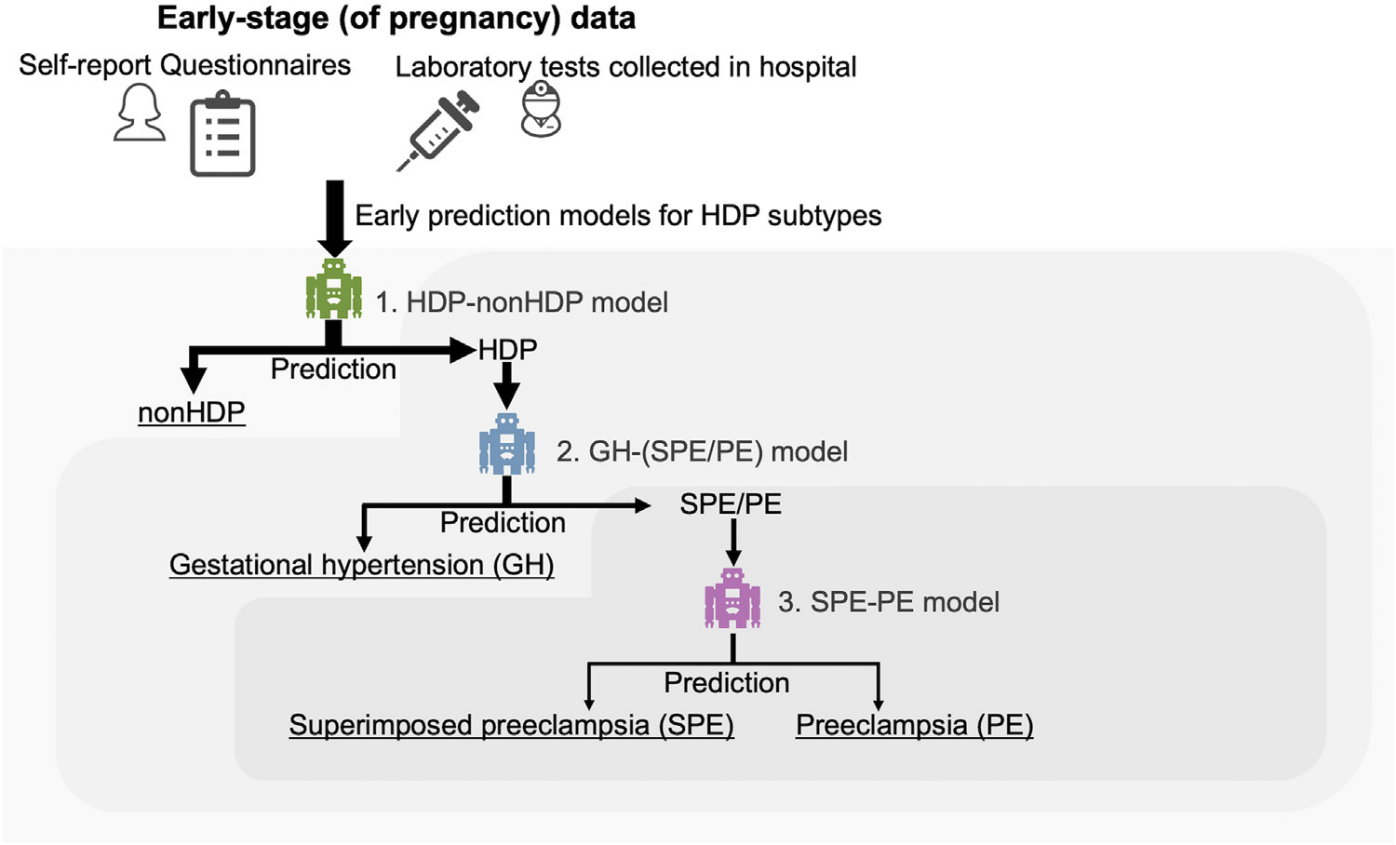
Early prediction models for four disease subtypes. We developed an early HDP prediction model using data collected in the early stage of pregnancy.

To avoid issues of the multi-class prediction models including the extreme label-imbalance, we developed the three binary prediction models in sequence according to the hierarchy of disease concepts as follows: 1) prediction model of HDP, including GH, PE and SPE among all subjects (called the HDP-nonHDP model); 2) prediction model of the presence of PU, including PE and SPE, among HDP subjects (called the GH-(SPE/PE) model); and 3) prediction model of PE among HDP subjects with PU (called the SPE-PE model) (Figure 1).

Mizuno. Early prediction of hypertensive disorders of pregnancy toward preventive early intervention. Am J Obstet Gynecol MFM 2024.

### Data preprocessing and model development

**Figure 2:**
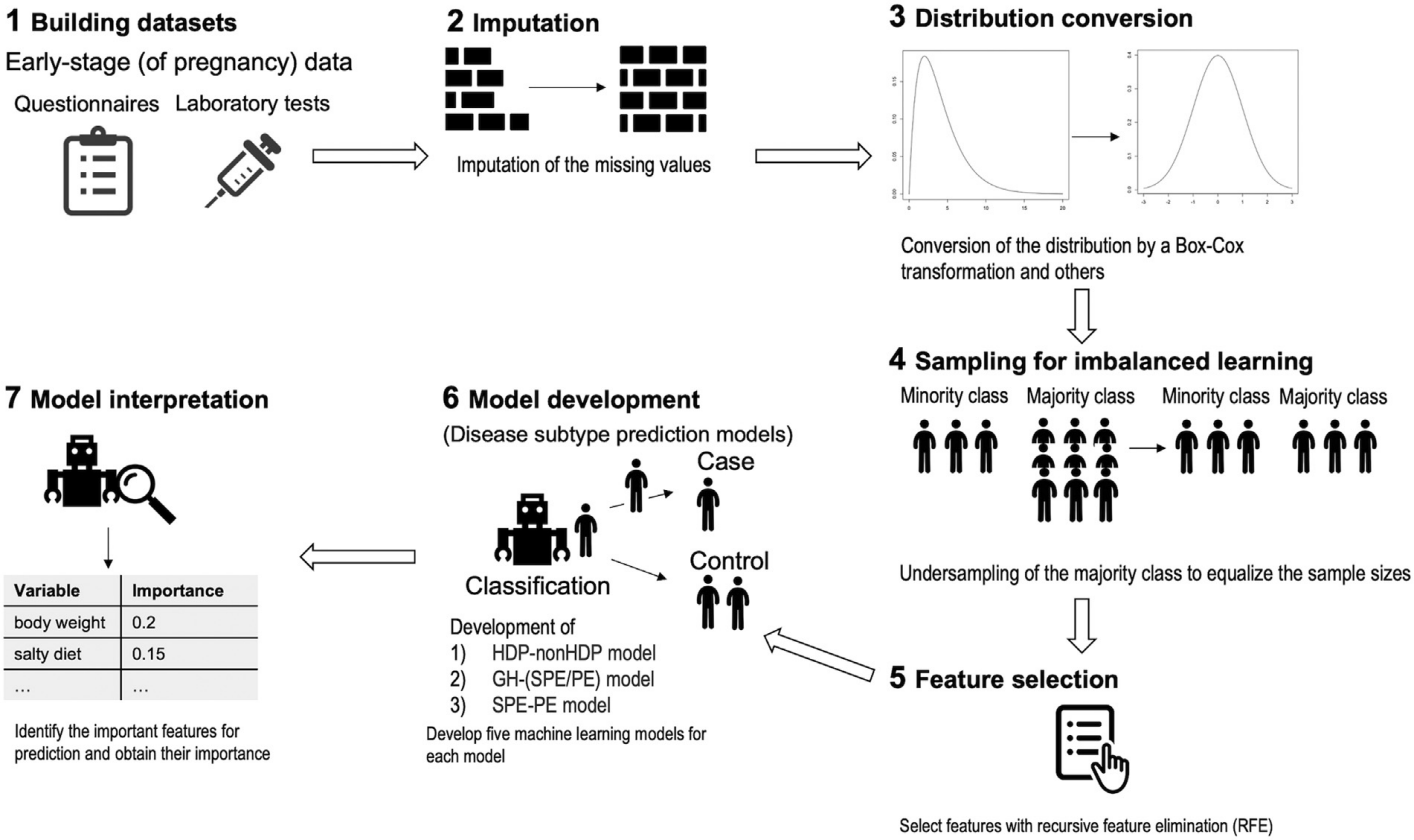
Overview of the data preprocessing and model development. In this study, we performed data preprocessing and built the following models: 1) building the datasets, 2) imputation of missing values, 3) distribution conversion, 4) sampling for imbalanced learning, 5) feature selection, 6) development of the prediction models, and 7) interpolation of the developed models. We investigated the optimal combination of methods for model development, such as distribution transformation, feature selection and model selection.

We performed data preprocessing and built the models as follows: 1) building the datasets, 2) preprocessing of data, 3) feature selection to maximize the performance of prediction, 4) development of the prediction models, and 5) interpolation of the developed models (Figure 2). The details of the processes are described below and in the supplementary methods.

Mizuno. Early prediction of hypertensive disorders of pregnancy toward preventive early intervention. Am J Obstet Gynecol MFM 2024.

#### Building the datasets

We built datasets using laboratory tests and questionnaires in early pregnancy for the early prediction models (Supplementary Table 1).

#### Preprocessing of data

We performed imputation of missing values, distribution conversion and sampling for preprocessing of data to improve the performance of the models. To impute missing values, we performed multivariate imputation by using chained equations (MICE).^20^ For distribution conversion, we used Box–Cox transformation, min-max scaling, and Box–Cox transformation plus min-max scaling. We performed sampling for imbalanced learning. We adapted undersampling of the majority class to balance the number of subjects between the majority and minority classes with the use of the NearMiss-1 algorithm^21^ for all models.

#### Feature selection

After sampling for imbalanced learning, we performed feature selection to avoid overfitting and improve the performance of the prediction model. We adapted recursive feature elimination (RFE)^22^ for feature selection.

#### Development of the machine learning model

To obtain a model with optimal performance, we built five machine learning models for all combinations of datasets and the two feature selection methods, as follows: 1) logistic regression (LR), 2) random forest (RF), 3) support vector machine (SVM), 4) deep neural network (DNN) and 5) XGBoost. The hyperparameters for DNN and their ranges of values are shown in Supplementary Table 2.

#### Interpretation of the machine learning model

In order to make the machine learning model explainable, we interpreted which features were important for learning for interpretable LR, RF, and XGBoost models among the developed models. For interpretation, both normalized absolute regression coefficients of the LR model by dividing by the sum of regression coefficients of all features, and Gini coefficients of the RF and XGBoost models were adapted as importance scores.

### Ethics

All participants consented to the gathering of their data. The study was approved by the ethics committee of the Tohoku Medical Megabank organization at Tohoku University (approval number: 2015-4-034, approved date: 10/27/2017). We obtained informed consent from all participants consented to the collection of their data. We obtained informed consent for all participants below the age of 16 from their parents or their legal guardian.

## Results

### Labeling subjects and building datasets

We identified 1781 (8.13%) HDP subjects by a phenotyping algorithm. These included 890 cases (4.06%) of GH, 304 cases (1.39%) of SPE and 587 cases (2.68%) of PE. The identified subtypes were used as the labels of our developed early prediction models. The numbers of HDP subjects for each dataset of the early prediction models are shown in Table.

**Table:** The number of features and subjects with HDP

Dataset	Number of features	Number of subjects	Number of subjects with HDP (n, %)	The mean gestational week of data collection (mean, SD)
Laboratory test data collected in the early stage of pregnancy	27	21,515	1,752 (8.14)	21.09 (6.6)
Questionnaires completed in the early stage of pregnancy	495	21,276	1,715 (8.06)	20.2 (7.9)

### High predictive performance of early prediction models for HDP-nonHDP

**Figure 3:**
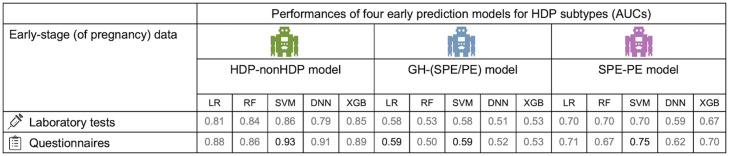
The AUC of the early disease prediction models. The AUCs of the early HDP-nonHDP, GH-(SPE/PE) and SPE-PE prediction models reached 0.93, 0.59 and 0.75, respectively.

We developed early disease prediction models using laboratory tests and questionnaire data in the early stage of pregnancy. The performances of all the prediction models are shown in Figure 3. We found that the maximum area under the receiver operating characteristic (ROC) curve (AUC) of those models reached 0.93 for the HDP-nonHDP model. The combination of data and machine learning models for the best prediction model of the HDP-nonHDP prediction model was questionnaires and SVM. These results clearly indicate that we have achieved to develop a precise early HDP prediction model based on only environmental factors.

Mizuno. Early prediction of hypertensive disorders of pregnancy toward preventive early intervention. Am J Obstet Gynecol MFM 2024.

### Eating habits are dominantly important for early prediction models

**Figure 4:**
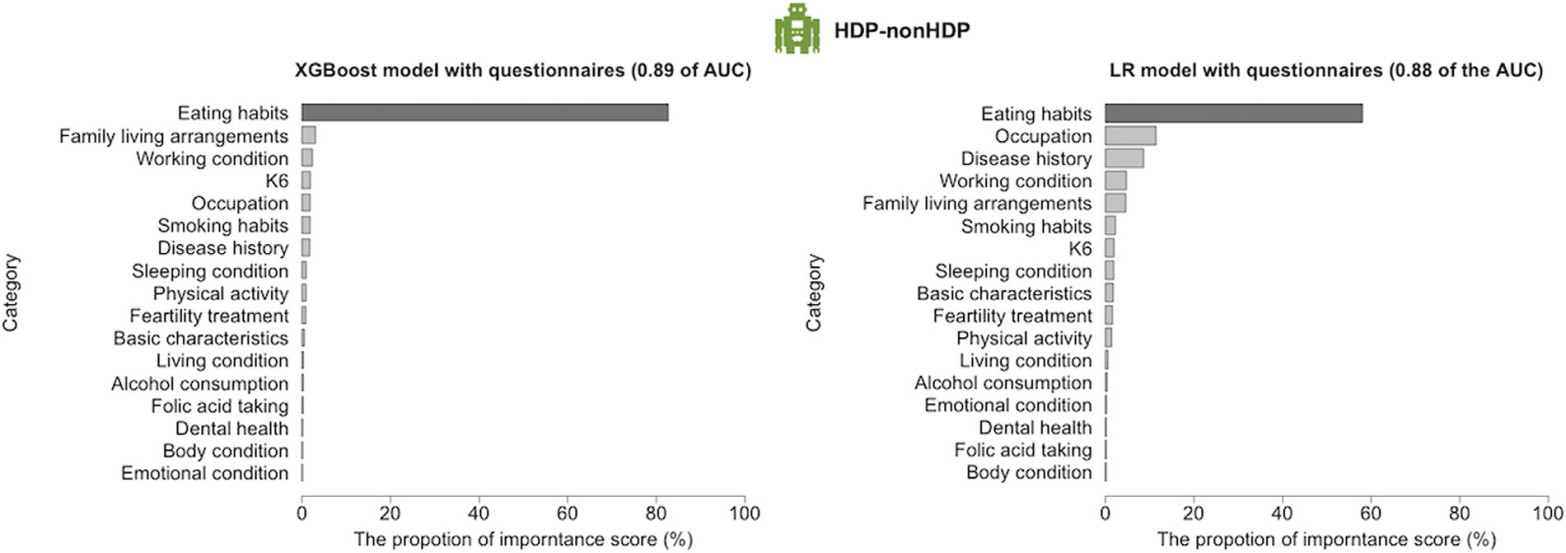
The influential features for prediction in HDP-nonHDP models. Among lifestyle factors, eating habits contributed 82.7% and 58.1% of all features in first and second highest performance models, respectively.

We interpreted the top two performances of the interpretable LR, RF, and XGBoost models among the five machine learning models of the HDP-nonHDP models by evaluating the importance of each feature in prediction. The AUC of the HDP-nonHDP models reached 0.89 and 0.88 in the XGBoost and LR models based on questionnaire data, respectively. In the interpretation results, eating habits (82.73 and 58.06%) showed dominantly high importance in the HDP-nonHDP model (Figure 4 and Supplementary Table 4). The high importance of these features may be reasonable because specific dietary patterns^23^ are known to increase blood pressure. This study is a remarkable achievement in that we can show that eating habits are more important in HDP-nonHDP models compared to many other environmental factors, such as smoking habits, which are known to affect blood pressure levels.^24^

Mizuno. Early prediction of hypertensive disorders of pregnancy toward preventive early intervention. Am J Obstet Gynecol MFM 2024.

### Modest performance models among the early disease prediction models

The GH-(SPE/PE) and SPE-PE models showed modest performance with AUCs of 0.59 and 0.75, respectively. These results are somewhat expected, and we surmise that the modest performance of the GH-(SPE/PE) and SPE-PE models might be caused by the large contribution of genetic factors in the development of PE.^10^ This point needs to be examined further.

### No further improvement of the early HDP prediction performances even with the additional data

We developed full-term disease prediction models to evaluate the possibility of performance improvement with additional full-term data that consisted of all the data collected during pregnancy (see the “Building the full-term datasets” section in the Supplementary methods and Supplementary Table 1) other than data used in early prediction models, and we found that the AUCs were increased up to 0.01, 0.08, and 0.07 for the HDP-nonHDP, GH-(SPE/PE) and SPE-PE prediction models, respectively (Supplementary Table 5). In the full-term models, we expected improvements in performance because of the enormous number of items from multiple data sources of full-term datasets; however, there were slight improvements compared with the corresponding early prediction models. These results indicate that the performances of early prediction models were saturated and adequate with data collected in the early stage of pregnancy (see the “Comparison of the performance of the early- and full-term prediction models” section in the Supplementary discussion).

## Discussion

### Principal findings

We developed early prediction models of HDP that achieved an AUC of 0.93 with comprehensive environmental factors from questionnaires completed earlier than HDP onset (mean of 20.2 gestational weeks for completion and 33.3 for onset). The interpretation of the developed models revealed that eating habits were especially important in the early prediction models.

### Results

Our early prediction models of HDP are the first generalized models using a population of 22,000, which is enormously larger and shows higher prediction performance than previous studies.^12^^,^^13^ Our early prediction model also exhibited higher (or optimal) performance with an AUC of 0.93 than other sophisticated multifactorial disease prediction models, such as a diabetes prediction model^25^ and a cardiovascular disease prediction model.^26^ The former model is an RF model with an AUC over 0.78 based on various environmental factors, laboratory tests, and physiological test data from over 8,000 Swedish people,^25^ and the latter model is a deep learning model combining an artificial neural network with a sparse autoencoder up to an F1-score of 0.90 based on the Framingham heart dataset.^26^

The highest-performance model was developed based on self-report questionnaire data in early pregnancy rather than laboratory tests. Of those, 495 lifestyle features were incorporated into the early prediction models, and then 375 features were used for the best HDP-nonHDP models. Thus, the sum of small effects from a large number of features contributes to the prediction of HDP like polyenviromic risk factors.^27^ In these polyenviromic risk factors, previously reported risk factors for HDP were included, such as eating habits,^23^ occupation,^28^ smoking habits,^24^ and weight before pregnancy.^29^ Among these risk factors, we found that the parts of well-known risk factors involving weight before pregnancy and smoking habits showed relatively lower importance, and interestingly, eating habits were highly influential for early prediction models of HDP.

### Clinical implications

One of the salient features of our prediction model is that the highest AUC of the early prediction model of HDP is achieved with a self-report questionnaire. Our results demonstrate the feasibility of the early prediction of HDP risk using lifestyle questionnaires filled before visiting hospital in clinical practice. Based on the prediction, clinicians are able to manage high risk patients of HDP and detect the signs of onsets early by closely monitoring patients to provide appropriate preventive measures. Clinicians are also able to practice preventive intervention in eating habits of patients to reduce onset risks for at least half of the cases of HDP who will subsequently develop GH, because eating habits were highly influential for early prediction of HDP.

### Research implications

Our study provided several further investigations.

1) Improvement of the PE and SPE prediction performance by adding further data such as genomic data because PE is known to be associated with genetic factors (the high estimated heritability of PE is up to 55%^10^)
2) Perform validation of the generalized performance of our developed model on subjects from other cohorts.
3) Development of the application for self-estimation of the risk of HDP and the prevention by early intervention in lifestyles to control the risk of HDP.

### Strengths and limitations

The first strength of our study is that we achieved to develop precise early prediction models of HDP based on self-controllable factors by pregnancy. This leads to implementations of early prediction, risk control, and early intervention of HDP at least subjects having GH. This is because of the high-performances of the HDP-nonHDP models and modest performances of both the GH-(SPE/PE) and SPE-PE models.

The second is the unbiased population of the dataset. Our dataset used in the models was collected from a large-scale general population. Because of this point, our early prediction models are generalized models. Furthermore, the large scale of our data reduces the effect of inherent potential self-report bias in the self-report questionnaire data.

The third is using comprehensive environmental factors because there are high hurdles to obtaining comprehensive environmental factors in EHR-based studies.

The first limitation of our study is that we could not implement genetic factors in early prediction models. This limitation may reduce the performance of the GH-(SPE/PE) model and the SPE-PE model.

The second limitation of this study is the lack of the external cohort based validation of the developed models because of the data availability. This study design led to the limitation in generalization performance in other populations.

## Conclusion

In this study, we developed high-performance early prediction models of HDP (called the HDP-nonHDP models) with lifestyle data in the early stage of pregnancy. These results clearly revealed the feasibility of clinical management of high-risk HDP patients and early intervention to provide appropriate preventive measures by detecting early onset signs through close monitoring. These results also revealed the possibility of self-estimation for the risk of HDP in early pregnancy, and the results will help to reduce the risk of all HDP. As for subtype prediction models of GH-(SPE/PE) and SPE-PE, these performances were not sufficient. Because of our achievements and the remaining challenges, this study serves as a basis for further studies on predictions, leading to improvements in PE and SPE prediction performance.

## CRediT authorship contribution statement

**Satoshi Mizuno:** Writing – original draft, Software, Methodology, Formal analysis, Conceptualization. **Satoshi Nagaie:** Writing – original draft, Software, Methodology, Formal analysis, Conceptualization. **Junichi Sugawara:** Writing – review & editing, Supervision, Conceptualization. **Gen Tamiya:** Writing – review & editing, Resources. **Taku Obara:** Writing – review & editing, Software. **Mami Ishikuro:** Writing – review & editing, Resources. **Shinichi Kuriyama:** Writing – review & editing, Resources. **Nobuo Yaegashi:** Writing – review & editing, Supervision, Conceptualization. **Hiroshi Tanaka:** Writing – review & editing, Resources. **Masayuki Yamamoto:** Writing – review & editing, Resources. **Soichi Ogishima:** Writing – review & editing, Project administration, Methodology, Conceptualization.

## Appendix Supplementary materials

Image, application 1

Image, image 2

Image, application 3

Image, application 4

Image, application 5

Image, application 6

Image, application 7

Image, application 8

Image, application 9

Image, application 10

## Footnotes

Supplementary material associated with this article can be found in the online version at doi:10.1016/j.xagr.2024.100383.
