# Supplementary material for: Early prediction of hypertensive disorders of pregnancy toward preventive early intervention
Source: AJOG Glob Rep. 2024 Jul 27;4(4):100383. doi: 10.1016/j.xagr.2024.100383 (PMC11550347; doi:10.1016/j.xagr.2024.100383)
Supplement: Supplementary file 1 [file mmc1.docx]

**Supplemental methods**

***Data sources***

We used the data collection of the BirThree Cohort Study as data sources. The data sources include laboratory tests, questionnaires, and medical records. Laboratory tests were not included in the medical records, but they were measured from specimens that were independently obtained in the BirThree Cohort. The medical record data of the first-visit interview and prenatal checkup were transcribed from the clinical records of usual care in clinics and hospitals by a trained genome medical research coordinator (GMRC). The overview of the items collected via the questionnaires is described in the “Assessments during the prenatal period” section of the previous report [1]. The list of items collected from the medical records and the settings of the prenatal checkup are described in the “Medical records” section of the previous report [1]. For the laboratory tests, items of the blood and urine tests that were collected included albumin, blood and urine creatinine levels, allergy tests, soluble fms-like tyrosine kinase-1 (sFlt1) values, placental growth factor (PLGF) values and hematological values.

***Details of the rules for identifying HDP subtypes***

HDP subtypes were defined by using blood pressure and urine dipstick tests at each prenatal checkup as follows. GH was defined as nonchronic hypertension with systolic blood pressure ≥ 140 mmHg or diastolic blood pressure ≥ 90 mmHg after 20 gestational weeks in the absence of PU. PE was defined as nonchronic hypertension, the absence of nephritis, systolic blood pressure ≥ 140 mmHg or diastolic blood pressure ≥ 90 mmHg after 20 gestational weeks, and PU ≥ 1+. SPE was defined as hypertension before 20 gestational weeks, complicated with PU after 20 gestational weeks.

***Building the full-term datasets***

In the building datasets, we excluded the following prenatal checkup variables, which were used in phenotyping: 1) blood pressure and PU after 32 weeks of pregnancy (which is the mean HDP onset time) were excluded from all of the models; 2) PU was excluded from the GH-SPE/PE model; and 3) blood pressure values before 20 weeks of pregnancy were excluded from the SPE-PE model. In this study, we built three sub-datasets from the prenatal checkup data with window sizes of 2 weeks, 5 weeks and 13 weeks called datasets 1, 2, and 3, respectively. For the development of the early prediction models, laboratory tests and questionnaires in early pregnancy were used. For the full-term prediction model, we used all of the available data except for the data used in the early prediction model including laboratory tests, questionnaires and medical records. The three types of prenatal checkup data with different window sizes were combined with all of the remaining data to create three concatenated datasets, which were named concatenated datasets 1, 2 and 3, respectively. In the creation of prenatal checkup datasets, the average value was taken for multiple measurements in the same gestational week.

***Imputation of missing values***

Before imputation with the MICE algorithm, we removed subjects with a high missing rate (greater than 70%) and variables with missing rates greater than 50% from datasets.

***Distribution conversion***

For distribution conversion, we used three methods, namely, Box-Cox transformation [2], min-max scaling, and Box-Cox transformation plus min-max scaling, to handle the many types of distributions in our dataset. In this study, we developed prediction models with the data generated by these three methods, and the best-performing model was used as the result.

***Sampling for imbalanced learning***

In this study, we compared the performances between the prediction models with undersampling using NearMiss-1 algorithm [3] and bootstrap aggregating (bagging), along with random sampling to evaluate the effect of sampling bias. For the development of the random sampling model, we used imblearn in Python 3. To evaluate whether sampling bias has occurred, we compared the performances between the prediction models with undersampling and bootstrap aggregating (bagging), along with random sampling. For the development of the bagging model, XGBoost [4] was utilized as the learning model The details of the learning models are described in the “Development of the machine learning model” section below.

***Feature selection***

In this study, we adapted recursive feature elimination (RFE) [5] using the RFE-CV function of scikit-learn [6] for the feature selection. RFE obtains a set of features with the highest prediction performance by weighting the features with an external estimator and repeatedly removing the variables with the smallest weights. For evaluate the suitable feature selection method, we compared the AUC of early prediction models using RFE with Hilbert-Schmidt independence criterion least absolute shrinkage and selection operator (HSIC-LASSO) [7] which obtain the set of independent feathers with labels and low redundancy in a nonlinear space. (see the "Comparison of the performance of feature selection methods" section below).

***Development of the machine learning model***

In this study, we used five machine learning models as follows: 1) logistic regression (LR), 2) random forest (RF) [8]. 3) support vector machine (SVM) [9], 4) deep neural network (DNN) and 5) XGBoost [4]. Scikit-learn [7] version 0.20 was used to develop the LR, RF and SVM models. Chainer [10, 11] version 5.0.0 was used to build the DNN model. The radial basis function (RBF) kernel was adapted for the SVM classifier. The parameters of the LR, RF, SVM and XGBoost models were optimized by maximizing the AUC with grid search. The optimized parameters of the DNN were obtained by maximizing the AUC with sequential model-based global optimization (SMBO) [12] using the hyperopt [13] library.

To avoid overfitting and to fix outliers in the datasets, we adopted internal-external cross-validation [14]. The final prediction performance was evaluated by the mean AUC of the iterations. In the internal-external cross-validation, 1/10 of the dataset was used as validation data, and the remaining 9/10 of the dataset was used for training the models using 10-fold cross-validation. After the training steps, we evaluated the prediction performance using the validation data. We performed 10 internal-external cross-validations with random sampling of the validation data to reduce the sampling bias of the validation data.

***Building the positive and negative controls***

We compared the prediction performance of the built models with positive and negative controls to detect fatal errors, such as extreme overfitting and underfitting, in the model building process. In this study, we created 100-1200 variables with high correlation coefficients of 0.60-0.95 with labels for positive controls. Negative controls were created by randomizing the labels of the datasets.

**Supplemental results**

***Number of selected features by feature selection***

We then performed feature selection using the HSIC-LASSO and the RFE, and the number of obtained features for each dataset and prediction tasks are shown in Supplemental Table 7.

***Comparison of the performance of feature selection methods***

The AUC of early prediction models using HSIC LASSO as feature selection methods reached to 0.90, 0.58 and 0.70 for HDP-nonHDP, GH-(SPE/PE) and SPE-PE prediction models, respectively and early prediction models with RFE reached to 0.93, 0.59 and 0.75, respectively (Supplemental table 5). This result showed that features obtained by RFE maximized the performance of early prediction models compared with HSIC LASSO, and we adopted RFE as feature selection method.

***Comparison of the performance between the early prediction model and bagging model***

To evaluate whether sampling bias has occurred, we compared the performances between undersampling and bootstrap aggregating (bagging). The maximum AUC of bagging were -0.33, -0.02 and ±0 for the HDP-nonHDP, GH-(SPE/PE) and SPE-PE prediction models, respectively, compared with the early prediction model with feature selection and undersampling (Supplemental Table 8). It showed that the prediction performances of feature selection plus undersampling were higher than bagging.

***Performance of positive controls and negative controls***

The maximum AUC for the positive and negative controls were 1.0 and 0.51, respectively (Supplemental Table 6).

**Supplemental discussion**

***Comparison of the performance of the early- and full-term prediction models***

Compared with the early prediction models, the AUC were increased up to 0.08 and 0.07 in GH-(SPE/PE) and SPE-PE prediction models, respectively in the full-term prediction models. The improvement of the GH-(SPE/PE) model with concatenated datasets may be caused by the inclusion of features reported as factors that are related to the development of PE in the full-term prediction model, such as the number of white blood cells [15] and values that are related to complete blood count (CBC) [16] which were not included in the early prediction models.

The reason for unavailability of these features in the early prediction model was caused by exclusion of these features from the dataset during data preprocessing because of the high missing rate. The high missing rate is due to the design of prenatal checkup in Japan, which is less visits in the early pregnancy and more visits in the late of pregnancy [17]. The unavailability of prenatal checkup data in the early stage of pregnancy is a limitation of this study. However, an AUC of 0.67 for the full-term GH-(SPE/PE) prediction model was not adequate for differentiating between the PE/SPE and GH subgroups in subjects with HDP. The improvement of SPE-PE prediction performance in the full-term prediction model may be caused by blood pressure after 20 weeks of pregnancy. In the early prediction model, laboratory tests and questionnaires (but not prenatal checkup data) were used for the developed model, and blood pressure values after 20 weeks, which were included in only the full-term prediction model, may have correlations with blood pressure values before 20 weeks of pregnancy, which is one of the classification criteria of SPE and PE in the clinical guidelines.

**Techniques for building the models**

***Imbalanced learning***

In this study, we adopted undersampling with the NearMiss-1 algorithm [18] for imbalanced learning. The NearMiss-1 algorithm is able to reduce the effect of sampling bias because it selects subjects who are near the border of the minority class. Compared with random sampling, sampling with the NearMiss-1 algorithm improves the performance of HDP-nonHDP prediction (Supplemental Tables 5 and 8).

***Feature selection***

For the feature selection of this study, we adapted both RFE, which is widely used in biomedical informatics studies [19, 20], and the HSIC-LASSO algorithm, which can select features that have nonlinear correlations between the labels and nonredundant features (Supplemental Table 3 and 7). In most of the prediction models, RFE outperformed the HSIC-LASSO algorithm (Supplemental Table 5). RFE selects the features that achieve the highest prediction performance. Due to the specification, the prediction models with RFE outperformed those with the HSIC-LASSO algorithm and have the possibility of overfitting for datasets through the selection of redundant features.

***Evaluation of overfitting***

In this study, we adapted both RFE, which is more likely to overfit the data, and the HSIC-LASSO algorithm, which is less likely to overfit the data, for feature selection and compared their prediction performances. The differences in the prediction performance between the models with RFE and the HSIC-LASSO algorithm were not drastic, with AUC differences of up to 0.03, 0.01 and 0.05 for the HDP-nonHDP, GH-SPE/PE and SPE-PE prediction models, respectively (Supplemental Table 5). These results indicate that strong overfitting to the dataset has not occurred in the feature selection process. Additionally, we performed internal-external cross-validation [14] to reduce overfitting and increase robustness to outliers in the dataset.

**References**

1 Sugawara J, Ishikuro M, Obara T, et al. Maternal baseline characteristics and perinatal outcomes: the Tohoku medical megabank project birth and three-generation cohort study. *J Epidemiol* 2020. doi:10.2188/jea.JE20200338.

2 Yeo IK, Johnson RA. A new family of power transformations to improve normality or symmetry. *Biometrika* 2000;87:954–9.

3 Mani I, Zhang I. kNN approach to unbalanced data distributions: a case study involving information extraction. In: Proceedings of Workshop on Learning from Imbalanced Datasets. Washington, DC: *ICML* 2003:1–7.

4 Chen T, Guestrin C. Xgboost: a scalable tree boosting system. In: Proceedings of the 22nd ACM SIGKDD International Conference on Knowledge Discovery and Data Mining. New York, NY: *Association for Computing Machinery* 2016:785–94.

5 Guyon I, Weston J, Barnhill S, et al. Gene selection for cancer classification using support vector machines. *Mach Learn* 2002;46:389–422.

6 Pedregosa F, Varoquaux G, Gramfort A, et al. Scikit-learn: machine learning in python. J *Mach Learn Res* 2011;12:2825–30.

7 Yamada M, Jitkrittum W, Sigal L, et al. High-dimensional feature selection by feature-wise kernelized lasso. *Neural Comput* 2014;26:185–207.

8 Breiman L. Random forests. *Mach Learn* 2001;45:5–32.

9 Cortes C, Vapnik V. Support-vector networks. *Mach Learn* 1995;20:273–97.

10 Tokui S, Oono K, Hido S, et al. Chainer: a next-generation open source framework for deep learning. *In: Proceedings of Workshop on Machine Learning Systems (LearningSys) in the 29th Annual Conference on Neural Information Processing Systems (NIPS)* 2015:1–6.

11 Akiba T, Fukuda K, Suzuki S. ChainerMN: scalable distributed deep learning framework. *arXiv*:171011351 2017.

12 Bergstra J, Bardenet R, Bengio Y, et al. Algorithms for hyper-parameter optimization. *In: 25th Annual Conference on Neural Information Processing Systems. Granada, Spain: Neural Information Processing Systems Foundation* 2011:2546–54.

13 Bergstra J, Yamins D, Cox D. Making a science of model search: hyperparameter optimization in hundreds of dimensions for vision architectures. *In: Proceedings of the 30 th International Conference on Machine Learning. Atlanta, Georgia: PMLR* 2013:115–23.

14 Steyerberg EW, Harrell Jr FE. Prediction models need appropriate internal, internal-external, and external validation. *J Clin Epidemiol* 2016;69:245.

15 Mtali YS, Lyimo MA, Luzzatto L, et al. Hypertensive disorders of pregnancy are associated with an inflammatory state: evidence from hematological findings and cytokine levels. *BMC Pregnancy Childbirth* 2019;19:237.

16 Elgari MM, Khabour OF, Alhag SM. Correlations between changes in hematological indices of mothers with preeclampsia and umbilical cord blood of newborns. *Clin Exp Hypertens* 2019;41:58–61.

17 H Minakami, T Maeda, T Fujii, et al. Guidelines for obstetrical practice in Japan: Japan Society Of Obstetrics and Gynecology (JSOG) and Japan Association of Obstetricians and Gynecologists (JAOG) 2014 edition. *J Obstet Gynaecol Res* 2014;6:1469-99.

18 Mani I, Zhang I. kNN approach to unbalanced data distributions: a case study involving information extraction. *In: Proceedings of Workshop on Learning from Imbalanced Datasets. Washington, DC: ICML* 2003:1–7.

19 Rehman RZU, Del Din S, Guan Y, et al. Selecting clinically relevant gait characteristics for classification of early parkinson’s disease: a comprehensive machine learning approach. *Sci Rep* 2019;9:17269

20 Wu X, Yuan X, Wang W, et al. Value of a machine learning approach for predicting clinical outcomes in young patients with hypertension. *Hypertension* 2020;75:1271–8.
