# Supplementary figures and images for: Early prediction of hypertensive disorders of pregnancy toward preventive early intervention
Source: AJOG Glob Rep. 2024 Jul 27;4(4):100383. doi: 10.1016/j.xagr.2024.100383 (PMC11550347; doi:10.1016/j.xagr.2024.100383)

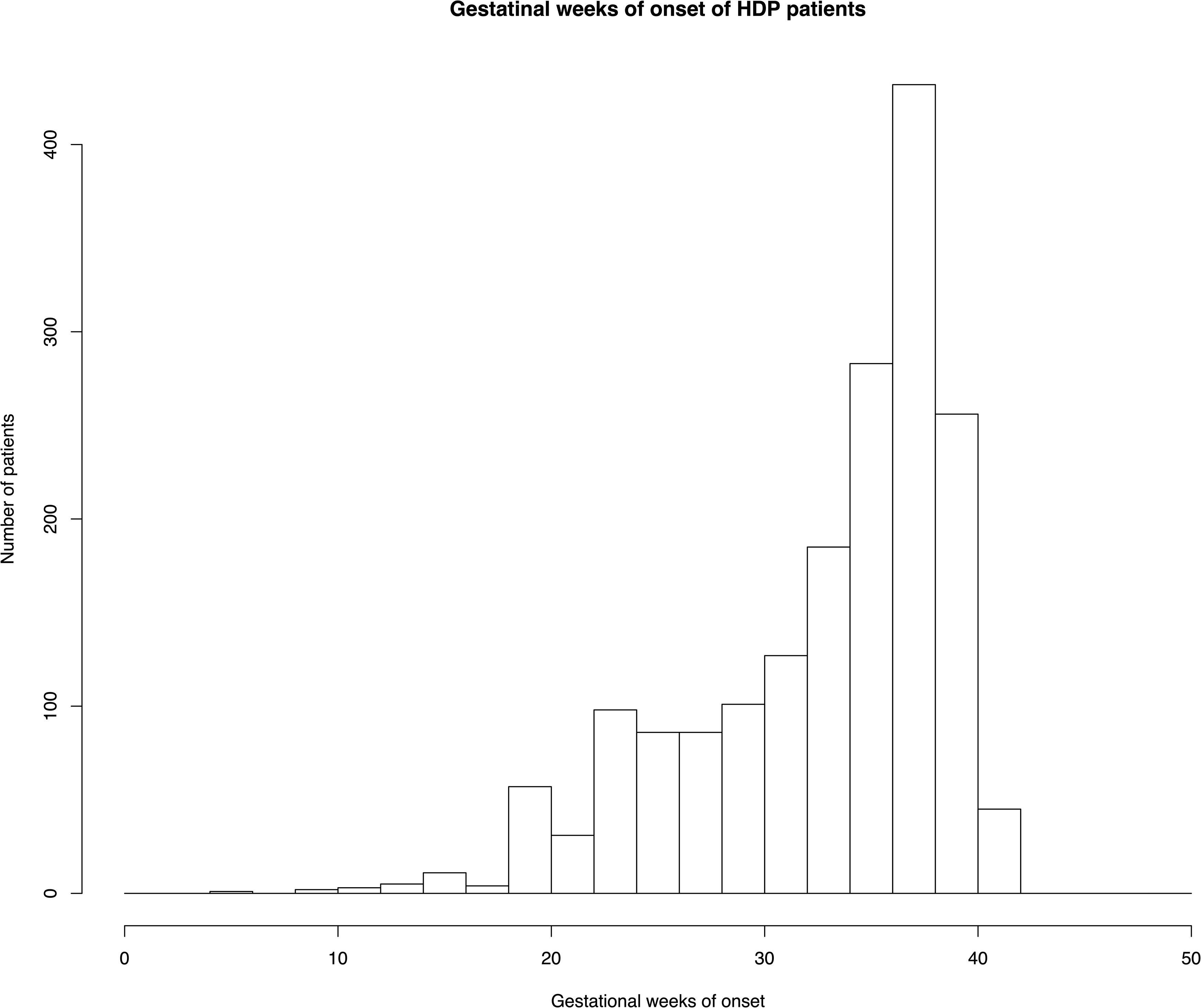

Supplement: Supplementary file 2 [file mmc2.jpg]
