# Supplementary material for: Early prediction of hypertensive disorders of pregnancy toward preventive early intervention
Source: AJOG Glob Rep. 2024 Jul 27;4(4):100383. doi: 10.1016/j.xagr.2024.100383 (PMC11550347; doi:10.1016/j.xagr.2024.100383)
Supplement: Supplementary file 3 [file mmc3.pdf]

Supplementary Table 1 : The datasets for both early and full-term prediction models

Datasets	The model in which dataset was used
Laboratory test data collected in the early stage of pregnancy	Early prediction model
Questionnaires completed in the early stage of pregnancy	Early prediction model
Questionnaires completed in the late stage of pregnancy	Full-term prediction model
Medical record of first visit interview	Full-term prediction model
Prenatal checkup data 2 (window sizes : 5 weeks)	Full-term prediction model
Prenatal checkup data 3 (window sizes : 13 weeks)	Full-term prediction model
Concatenated dataset 1 (window sizes : 2 weeks)	Full-term prediction model
Concatenated dataset 2 (window sizes : 5 weeks)	Full-term prediction model
Concatenated dataset 3 (window sizes : 13 weeks)	Full-term prediction model
