# Supplementary material for: Early prediction of hypertensive disorders of pregnancy toward preventive early intervention
Source: AJOG Glob Rep. 2024 Jul 27;4(4):100383. doi: 10.1016/j.xagr.2024.100383 (PMC11550347; doi:10.1016/j.xagr.2024.100383)
Supplement: Supplementary file 4 [file mmc4.pdf]

Supplementary Table 2 : The hyperparameters for DNN models

Hyperparameters	Range of parameters
Number of layers	3-5
Number of units for each layer	10-50
Activation function	Relu/sigmoid
Optimizer	Adam/AdaDelta/MomentumSGD
Batchsize	10-1000
epoch	5-20
lr	0.005-0.02
Using dropout	True/False
Dropout rate if using dropout	0.2/0.8
