# Supplementary material for: Early prediction of hypertensive disorders of pregnancy toward preventive early intervention
Source: AJOG Glob Rep. 2024 Jul 27;4(4):100383. doi: 10.1016/j.xagr.2024.100383 (PMC11550347; doi:10.1016/j.xagr.2024.100383)
Supplement: Supplementary file 5 [file mmc5.pdf]

Supplementary Table 3 : The proportion of datasets

HDP-nonHDP model

Datasets	Number of features	Number of subjects	Number of HDP subjects (n, %)
Laboratory test data collected in the early stage of pregnancy	27	22066	1752 (7.94)
Questionnaires completed in the early stage of pregnancy	495	21815	1715 (7.86)
Questionnaires completed in the late stage of pregnancy	565	21497	1687 (7.85)
Medical record of first visit interview	130	22441	1779 (7.93)
Prenatal checkup data 2	975	18548	1485 (8.01)
Prenatal checkup data 3	1131	21949	1746 (7.95)
Concatenated dataset 1	1685	21376	1561 (7.30)
Concatenated dataset 2	2150	21794	1672 (7.67)
Concatenated dataset 3	2347	22180	1761 (7.94)

GH-(SPE/PE) model

Datasets	Number of features	Number of subjects	Number of subjects with smaller numbers in SPE/PE (n, %)
Laboratory test data collected in the early stage of pregnancy	27	22066	872 (3.95)
Questionnaires completed in the early stage of pregnancy	495	21815	856 (3.92)
Questionnaires completed in the late stage of pregnancy	565	21497	840 (3.91)
Medical record of first visit interview	130	22441	889 (3.96)
Prenatal checkup data 2	952	17923	700 (3.91)
Prenatal checkup data 3	1101	21892	872 (3.98)
Concatenated dataset 1	1669	21369	832 (3.89)
Concatenated dataset 2	2123	21786	854 (3.92)
Concatenated dataset 3	2317	22134	879 (3.97)

SPE-PE model

Datasets	Number of features	Number of subjects	Number of SPE subjects (n, %)
Laboratory test data collected in the early stage of pregnancy	27	22066	300 (1.36)
Questionnaires completed in the early stage of pregnancy	495	21815	296 (1.36)
Questionnaires completed in the late stage of pregnancy	565	21497	293 (1.36)
Medical record of first visit interview	130	22441	304 (1.35)
Prenatal checkup data 2	951	18602	262 (1.41)
Prenatal checkup data 3	1091	21929	300 (1.37)
Concatenated dataset 1	1673	21392	290 (1.36)
Concatenated dataset 2	2126	21798	298 (1.37)
Concatenated dataset 3	2307	22157	302 (1.36)
