# Supplementary material for: Early prediction of hypertensive disorders of pregnancy toward preventive early intervention
Source: AJOG Glob Rep. 2024 Jul 27;4(4):100383. doi: 10.1016/j.xagr.2024.100383 (PMC11550347; doi:10.1016/j.xagr.2024.100383)
Supplement: Supplementary file 6 [file mmc6.pdf]

Supplementary Table 4 : The feature importances of the early prediction models based on health assesment data

HDP-nonHDP model

Questionnaires completed in the early stage of pregnancy, RFE, XGBOOST, AUC=0.89		
Feature name	Category	Importance score
Is your daily food intake in the past year higher than the reference amount? (reference amount: 1 piece of sea bream, 70g)	Eating habits	0.05
Is your daily food intake in the past year higher than the reference amount? (reference amount: 1 medium leaf of lettuce, 10g)	Eating habits	0.03
Is your daily food intake in the past year higher than the reference amount? (reference amount: 80 g per horse mackerel or sardine)	Eating habits	0.03
Is your daily food intake in the past year higher than the reference amount? (reference amount: 1/4 papaya, 50g)	Eating habits	0.02
Is your daily food intake in the past year higher than the reference amount? (reference amount: 1/4 of an onion, 50g)	Eating habits	0.02
Is your daily food intake in the past year higher than the reference amount? (reference amount: 6 pea pods, 30g)	Eating habits	0.02
Is your daily food intake in the past year higher than the reference amount? (reference amount: 2 tablespoons of shirasu-boshi, 10g)	Eating habits	0.02
Is your daily food intake in the past year higher than the reference amount? (reference amount: 1/3 potato, 50g)	Eating habits	0.02
Is your daily food intake in the past year higher than the reference amount? (reference amount:1/4 bag bean sprouts 25g)	Eating habits	0.02
Is your daily food intake in the past year higher than the reference amount? (reference amount:4 slices of Buri/Hamachi sashimi(60g))	Eating habits	0.01
Is your daily food intake in the past year higher than the reference amount? (reference amount: 1/3 bunch of shunugiku, 30g)	Eating habits	0.01
Is your daily food intake in the past year higher than the reference amount? (reference amount:1/3 cucumber 30g)	Eating habits	0.01
Is your daily food intake in the past year higher than the reference amount? (reference amount: 2 chives, 20g)	Eating habits	0.01
Is your daily food intake in the past year higher than the reference amount? (reference amount:1 komatsuna plant, 20g)	Eating habits	0.01
Is your daily food intake in the past year higher than the reference amount? (reference amount : apple pear 1/2 80g)	Eating habits	0.01
Is your daily food intake in the past year higher than the reference amount? (reference amount:1/3 of an octopus leg (50g))	Eating habits	0.01
Is your daily food intake in the past year higher than the reference amount? (reference amount: 1 tablespoon of dressing, 10g)	Eating habits	0.01
Is your daily food intake in the past year higher than the reference amount? (reference amount: 1/4 of a shimeji mushroom, 20g)	Eating habits	0.01
Is your daily food intake in the past year higher than the reference amount? (reference amount: 10 clams or clam shells, 20g)	Eating habits	0.01

Questionnaires completed in the early stage of pregnancy, RFE, LR, AUC=0.88		
Feature name	Category	Importance score
Height of body	Basic characteristics	0.02
Diagnosis received during past pregnancy: Hypertensive disorder of pregnancy	Disease history	0.01
Is your daily food intake in the past year higher than the reference amount? (reference amount: 1/4 of an onion, 50g)	Eating habits	0.01
Is your daily food intake in the past year higher than the reference amount? (reference amount: 1/4 papaya, 50g)	Eating habits	0.01
Have your eating habits changed significantly in the past 5 years?	Eating habits	0.01
Is your daily food intake in the past year higher than the reference amount? (reference amount: 1/3 of a leaf of Chinese cabbage, 30g)	Eating habits	0.01
Is your daily food intake in the past year higher than the reference amount? (reference amount: 1/3 potato, 50g)	Eating habits	0.01
Is your daily food intake in the past year higher than the reference amount? (reference amount:1 piece of dried fish, 50g))	Eating habits	0.01
Disease History: malformation of the uterus	Disease history	0.01
Is your daily food intake in the past year higher than the reference amount? (reference amount: 1 piece of sea bream, 70g)	Eating habits	0.01
Disease History: uterine myoma	Disease history	0.01
Occupation: occupations that do not fit into any of the above categories	Occupation	0.01
Is your daily food intake in the past year higher than the reference amount? (reference amount: 80 g per horse mackerel or sardine)	Eating habits	0.01
Is your daily food intake in the past year higher than the reference amount? (reference amount: 8g of jam or marmalade per slice of bread)	Eating habits	0.01
Is your daily food intake in the past year higher than the reference amount? (reference amount: 2 tablespoons of shirasu-boshi, 10g)	Eating habits	0.01
Is your daily food intake in the past year higher than the reference amount? (reference amount: 1 medium leaf of lettuce, 10g)	Eating habits	0.01
Are you living with your mother?	Household composition	0.01
Are you living with your partner's siblings?	Household composition	0.01
Have you undergone fertility treatment?	Feartility treatment	0.01

Is your daily food intake in the past year higher than the reference amount? (reference amount: 1/2 sheet of Koya tofu/frozen bean curd 60g)	Eating habits	0.01
Is your daily food intake in the past year higher than the reference amount? (reference amount: 1/3 of a leaf of Chinese cabbage, 30g)	Eating habits	0.01
Is your daily food intake in the past year higher than the reference amount? (reference amount: 3 bunches of broccoli, 30g)	Eating habits	0.01
Is your daily food intake in the past year higher than the reference amount? (reference amount: 10 large grapes, 100g)	Eating habits	0.01
Is your daily food intake in the past year higher than the reference amount? (reference amount: 1 shiitake mushroom, 20g)	Eating habits	0.01
Is your daily food intake in the past year higher than the reference amount? (reference amount: 1 eggplant, 60g)	Eating habits	0.01
Is your daily food intake in the past year higher than the reference amount? (reference amount: 8g of jam or marmalade per slice of bread)	Eating habits	0.01
Is your daily food intake in the past year higher than the reference amount? (reference amount: 1/4 burdock root, 40g)	Eating habits	0.01
Is your daily food intake in the past year higher than the reference amount? (reference amount: 1/4 of a melon, 60g)	Eating habits	0.01
Is your daily food intake in the past year higher than the reference amount? (reference amount: 4 slices of bonito and tuna, 60g)	Eating habits	0.01
Is your daily food intake in the past year higher than the reference amount? (reference amount: 50g chicken cubes)	Eating habits	0.01
Is your daily food intake in the past year higher than the reference amount? (reference amount:3 cubes of stewed beef(50g))	Eating habits	0.01
Is your daily food intake in the past year higher than the reference amount? (reference amount:1 slice of bacon 20g)	Eating habits	0.01
Is your daily food intake in the past year higher than the reference amount? (reference amount: 1 teaspoon of sauce, 5 g)	Eating habits	0.01
Have your eating habits changed significantly in the past 5 years?	Eating habits	0.01
Is your daily food intake in the past year higher than the reference amount? (reference amount: 1 taro, 30g)	Eating habits	0.01
Is your daily food intake in the past year higher than the reference amount? (reference amount:1/8 watermelon 120g)	Eating habits	0.01
Is your daily food intake in the past year higher than the reference amount? (reference amount: 3 pieces of fried chicken, 50g)	Eating habits	0.01
Is your daily food intake in the past year higher than the reference amount? (reference amount: 1/2 skewer of eel, 50g)	Eating habits	0.01
Is your daily food intake in the past year higher than the reference amount? (reference amount: 1/4 of enoki mushrooms, 20g)	Eating habits	0.01
Is your daily food intake in the past year higher than the reference amount? (reference amount:1/4 green onion (20g))	Eating habits	0.01
Is your daily food intake in the past year higher than the reference amount? (reference amount:(5 slices of stir-fried chicken(60g))	Eating habits	0.01
During the last 30 days, about how often did you feel hopeless?	K6	0.01

Fertility Treatment: In Vitro Fertilization	Feartility treatment	0.01
Are you living with your partner's father?	Household composition	0.01
Occupation: chef	Occupation	0.01
Occupation: service professional	Occupation	0.01
Is your daily food intake in the past year higher than the reference amount? (reference amount: 8g margarine for 1 slice of bread)	Eating habits	0.01
Is your daily food intake in the past year higher than the reference amount? (reference amount: 1/3 bunch of shunugiku, 30g)	Eating habits	0.01
Is your daily food intake in the past year higher than the reference amount? (reference amount: 80 g per horse mackerel or sardine)	Eating habits	0.01
Is your residence or workplace a noisy environment?	Living condition	0.01
Is your daily food intake in the past year higher than the reference amount? (reference amount: 6 pea pods, 30g)	Eating habits	0.01
Is your daily food intake in the past year higher than the reference amount? (reference amount: 1/2 sheet of Koya tofu/frozen bean curd 60g)	Eating habits	0.01
Is your daily food intake in the past year higher than the reference amount? (reference amount: 20 peanuts, 20g)	Eating habits	0.01
Is your daily food intake in the past year higher than the reference amount? (reference amount: about 150g per steak)	Eating habits	0.01
Is your daily food intake in the past year higher than the reference amount? (reference amount:1/4 bag bean sprouts 25g)	Eating habits	0.01
How often do you consume soy milk?	Eating habits	0.01
Is your daily food intake in the past year higher than the reference amount? (reference amount: 10 clams or clam shells, 20g)	Eating habits	0.01
Is your daily food intake in the past year higher than the reference amount? (reference amount: 1 medium egg, 50g)	Eating habits	0.01
Did you work in a hot place that made you sweat heavily?	Working condition	0.01
Total sleeping hours	Sleeping condition	0.01
Did you go in and out of the refrigerator or freezer in your daily life or work?	Working condition	0.01
How often do you consume peach?	Eating habits	0.01
Is your daily food intake in the past year higher than the reference amount? (reference amount:1/3 of an octopus leg (50g))	Eating habits	0.01
Have you had mumps in the past 6 months?	Disease history	0.01
Is your daily food intake in the past year higher than the reference amount? (reference amount: 200cc of low-fat milk, 1 bottle)	Eating habits	0.01

Is your daily food intake in the past year higher than the reference amount? (reference amount: 1/2 apple, 85g)	Eating habits	0.01
Is your daily food intake in the past year higher than the reference amount? (reference amount:1/4 tomato (50g))	Eating habits	0.01
Frequency of passive smoking in the year before pregnancy	Smoking habits	0.00
Are you living with your partner's father?	Household composition	0.00
Is your daily food intake in the past year higher than the reference amount? (reference amount: 80 g per horse mackerel or sardine)	Eating habits	0.00
Is your daily food intake in the past year higher than the reference amount? (reference amount: 1 piece of salmon or trout fillet, 70g)	Eating habits	0.00
Is your daily food intake in the past year higher than the reference amount? (reference amount: 1 teaspoon ketchup, 5g)	Eating habits	0.00
Diagnosis received during past pregnancy: Hypertensive disorder of pregnancy	Disease history	0.00
Is your daily food intake in the past year higher than the reference amount? (reference amount: 1/2 of a peach, 65g)	Eating habits	0.00
Are you living with your mother?	Household composition	0.00
Is your daily food intake in the past year higher than the reference amount? (reference amount: 15g per piece of loin ham)	Eating habits	0.00
Is your daily food intake in the past year higher than the reference amount? (reference amount:3 thin slices of stir-fried pork(60g))	Eating habits	0.00
Is your daily food intake in the past year higher than the reference amount? (reference amount: 1/8 of a pineapple, 130g)	Eating habits	0.00
Have you undergone fertility treatment?	Feartility treatment	0.00
Is your daily food intake in the past year higher than the reference amount? (reference amount: 2 slices of fish paste, 20g)	Eating habits	0.00
Occupation: service professional	Occupation	0.00
Are you living with your partner's mother?	Household composition	0.00
Height of body	Basic characteristics	0.00
Is your daily food intake in the past year higher than the reference amount? (reference amount:1 small plate of pickles (cucumbers) 30 g))	Eating habits	0.00
Is your daily food intake in the past year higher than the reference amount? (reference amount:(3 cubes of stewed pork(50g))	Eating habits	0.00
Is your daily food intake in the past year higher than the reference amount? (reference amount: 2 slices of boiled pork, 40g)	Eating habits	0.00
Fertility Treatment: In Vitro Fertilization	Feartility treatment	0.00
Is your daily food intake in the past year higher than the reference amount? (reference amount: 8g margarine for 1 slice of bread)	Eating habits	0.00

Is your daily food intake in the past year higher than the reference amount? (reference amount:2 pieces of grilled chicken(70g))	Eating habits	0.01
Is your daily food intake in the past year higher than the reference amount? (reference amount: 1 tablespoon of dressing, 10g)	Eating habits	0.01
Smoking habit	Smoking habits	0.01
How often do you consume apple pear?	Eating habits	0.01
Is your daily food intake in the past year higher than the reference amount? (reference amount: 1/4 of a melon, 60g)	Eating habits	0.01
How long does it take to fall asleep?	Sleeping condition	0.01
Is your daily food intake in the past year higher than the reference amount? (reference amount: 3 pieces of fried chicken, 50g)	Eating habits	0.01
How often do you take breakfast?	Eating habits	0.00
Frequency of passive smoking in the year before pregnancy	Smoking habits	0.00
Are you living with your partner's mother?	Household composition	0.00
Is your daily food intake in the past year higher than the reference amount? (reference amount:1 slice of bread, 6 slices(60 g))	Eating habits	0.00
Do you live with anyone other than the above?	Household composition	0.00
Is your daily food intake in the past year higher than the reference amount? (reference amount:1 small dish of pickled vegetables (eggplant)(30 g))	Eating habits	0.00
Disease History: dysmenorrhea	Disease history	0.00
How often do you consume source?	Eating habits	0.00
Reason for change in eating habits: Abnormalities in tests	Eating habits	0.00
Is your daily food intake in the past year higher than the reference amount? (reference amount : apple pear 1/2 80g)	Eating habits	0.00
Do you add suger to your tea?	Eating habits	0.00
During the last 30 days, about how often did you feel restless or fidgety?	K6	0.00
During the last 30 days, about how often did you feel worthless?	K6	0.00
Is your daily food intake in the past year higher than the reference amount? (reference amount:1 banana (75g))	Eating habits	0.00
Is your daily food intake in the past year higher than the reference amount? (reference amount: 1/2 medium cabbage leaf, 30g)	Eating habits	0.00
Were there any days in the week before your pregnancy when you walked continuously for more than 10 minutes?	Physical activity	0.00

Is your daily food intake in the past year higher than the reference amount? (reference amount:1 piece of dried fish, 50g))	Eating habits	0.00
Is your daily food intake in the past year higher than the reference amount? (reference amount: 3 slices of squid, 50g)	Eating habits	0.00
Is your daily food intake in the past year higher than the reference amount? (reference amount:1 small bowl of wakame seaweed/kelp 20g)	Eating habits	0.00
How often do you take breakfast?	Eating habits	0.00
K6 score	K6	0.00
Is your daily food intake in the past year higher than the reference amount? (reference amount:2 pcs Taisho shrimp(40g))	Eating habits	0.00
Do you live with anyone other than the above?	Household composition	0.00
Did you work in a hot place that made you sweat heavily?	Working condition	0.00
Is your daily food intake in the past year higher than the reference amount? (reference amount:1/6 sweet potatoes (40g))	Eating habits	0.00
Is your daily food intake in the past year higher than the reference amount? (reference amount: 2 cookies, 25g)	Eating habits	0.00
Did you go in and out of the refrigerator or freezer in your daily life or work?	Working condition	0.00
Is your daily food intake in the past year higher than the reference amount? (reference amount: 1/2 tbsp mayonnaise, 7g)	Eating habits	0.00
Is your daily food intake in the past year higher than the reference amount? (reference amount: 8g butter for 1 slice of bread)	Eating habits	0.00
Passive smoking in the year before pregnancy hours per day	Smoking habits	0.00
Is your daily food intake in the past year higher than the reference amount? (reference amount: 2 heads of spinach, 50g)	Eating habits	0.00
During the last 30 days, about how often did you feel so depressed that nothing could cheer you up?]	K6	0.00
Is your residence or workplace a noisy environment?	Living condition	0.00
Passive smoking up to Junior high school hours per day	Smoking habits	0.00
Is your daily food intake in the past year higher than the reference amount? (reference amount: 1/2 of other citrus fruits, 75g)	Eating habits	0.00
Is your daily food intake in the past year higher than the reference amount? (reference amount:1 slice of cheese (20g))	Eating habits	0.00
Disease History: uterine myoma	Disease history	0.00
Is your daily food intake in the past year higher than the reference amount? (reference amount:1 slice of small shortcake 70g)	Eating habits	0.00
Is your daily food intake in the past year higher than the reference amount? (reference amount: 5 sheets of seasoned seaweed, 2g)	Eating habits	0.00
Is your daily food intake in the past year higher than the reference amount? (reference amount: 1 small cup of natto, 50g)	Eating habits	0.00

Reason for change in eating habits: got sick	Eating habits	0.00
Occupation: sales position	Occupation	0.00
Occupation: product sales	Occupation	0.00
Disease History: endometriosis	Disease history	0.00
Occupation: social welfare specialists	Occupation	0.00
Is your daily food intake in the past year higher than the reference amount? (reference amount: 3 bunches of broccoli, 30g)	Eating habits	0.00
How often do you consume soba?	Eating habits	0.00
Is your daily food intake in the past year higher than the reference amount? (reference amount: 1 shiitake mushroom, 20g)	Eating habits	0.00
Occupation: other health care professionals	Occupation	0.00
How often do you consume milk?	Eating habits	0.00
Reason for change in eating habits: Change in taste	Eating habits	0.00
Are you living with your partner?	Household composition	0.00
Did you handle hair dye at work at least once a month during this pregnancy?	Working condition	0.00
Have you taken sleeping pills more than three times a week in the past month?	Sleeping condition	0.00
Is your daily food intake in the past year higher than the reference amount? (reference amount: 8g butter for 1 slice of bread)	Eating habits	0.00
Is your daily food intake in the past year higher than the reference amount? (reference amount:(5 slices of stir-fried chicken(60g))	Eating habits	0.00
Diagnosis received during past pregnancy: threatened preterm labor	Disease history	0.00
Frequency of consumption of miso soup	Eating habits	0.00
Have you had the flu in the past 6 months?	Disease history	0.00
Passive smoking up to Junior high school hours per day	Smoking habits	0.00
How often do you consume sweet potato?	Eating habits	0.00
Diagnosis received during past pregnancy: Other obstetric and gynecological diseases	Disease history	0.00
How often do you consume yellowtail amberjack?	Eating habits	0.00
Is your daily food intake in the past year higher than the reference amount? (reference amount: 1/4 burdock root, 40g)	Eating habits	0.00

Is your daily food intake in the past year higher than the reference amount? (reference amount:1/4 tofu 75g)	Eating habits	0.00
Did you handle chlorine bleach and disinfectant at work at least once a month during this pregnancy?	Working condition	0.00
Is your daily food intake in the past year higher than the reference amount? (reference amount: 1/2 medium cabbage leaf, 30g)	Eating habits	0.00
During the last 30 days, about how often did you feel restless or fidgety?	K6	0.00
Occupation: chef	Occupation	0.00
Is your daily food intake in the past year higher than the reference amount? (reference amount:2 pieces of grilled chicken(70g))	Eating habits	0.00
Are you living with your child?	Household composition	0.00
Is your daily food intake in the past year higher than the reference amount? (reference amount:1/4 of cod roe(25g))	Eating habits	0.00
Is your daily food intake in the past year higher than the reference amount? (reference amount:1/4 canned tuna 20g)	Eating habits	0.00
How often do you consume braised pork?	Eating habits	0.00
During the last 30 days, about how often did you feel nervous?	K6	0.00
How many people, including yourself, live in the house?	Household composition	0.00
How often do you consume simmered chicken?	Eating habits	0.00
Is your daily food intake in the past year higher than the reference amount? (reference amount: 200cc of low-fat milk, 1 bottle)	Eating habits	0.00
Is your daily food intake in the past year higher than the reference amount? (reference amount: 2 mandarins, 140g)	Eating habits	0.00
How often do you consume ramen?	Eating habits	0.00
Did you handle hair dye at work at least once a month during this pregnancy?	Working condition	0.00
Frequency of consumption of miso soup	Eating habits	0.00
During this pregnancy, did you handle permanent marker at work at least once a month?	Working condition	0.00
How often do you consume milk?	Eating habits	0.00
Smoking habit	Smoking habits	0.00
Were there any days in the week before your pregnancy when you walked continuously for more than 10 minutes?	Physical activity	0.00
Is your daily food intake in the past year higher than the reference amount? (reference amount:3 thin slices of stir-fried beef(60g))	Eating habits	0.00
Disease History: endometriosis	Disease history	0.00
Is your daily food intake in the past year higher than the reference amount? (reference amount: 5 strawberries, 75g)	Eating habits	0.00
Is your daily food intake in the past year higher than the reference amount? (reference amount:1/4 carrot (50g))	Eating habits	0.00
Are you living with your partner's siblings?	Household composition	0.00

Is your daily food intake in the past year higher than the reference amount? (reference amount: 2 slices of boiled pork, 40g)	Eating habits	0.00
Is your daily food intake in the past year higher than the reference amount? (reference amount: 5 sheets of seasoned seaweed, 2g)	Eating habits	0.00
Is your daily food intake in the past year higher than the reference amount? (reference amount:1/4 tomato (50g))	Eating habits	0.00
How often do you consume grape?	Eating habits	0.00
Are you living with your father?	Household composition	0.00
During the last 30 days, about how often did you feel hopeless?	K6	0.00
Diagnosis received during past pregnancy: placenta abruption	Disease history	0.00
How often do you consume braised pork?	Eating habits	0.00
Is your daily food intake in the past year higher than the reference amount? (reference amount: 2 chives, 20g)	Eating habits	0.00
How often do you consume roast ham?	Eating habits	0.00
Is your daily food intake in the past year higher than the reference amount? (reference amount:4 slices of Buri/Hamachi sashimi(60g))	Eating habits	0.00
Disease History: Ovarian Tumor/Ovarian Cyst	Disease history	0.00
Did you handle chlorine bleach and disinfectant at work at least once a month during this pregnancy?	Working condition	0.00
Occupation: researcher	Occupation	0.00
Reason for changing eating habits: thought it was good for my health	Eating habits	0.00
Is your daily food intake in the past year higher than the reference amount? (reference amount: 4 slices of bonito and tuna, 60g)	Eating habits	0.00
How often do you consume pork soup?	Eating habits	0.00
Is your daily food intake in the past year higher than the reference amount? (reference amount: 1/4 of enoki mushrooms, 20g)	Eating habits	0.00
Occupation: other technicians	Occupation	0.00
Disease History: Other obstetric and gynecological diseases	Disease history	0.00
Have you had rubella in the past six months?	Disease history	0.00
Occupation: information processing and telecommunications rngineer	Occupation	0.00
Is your daily food intake in the past year higher than the reference amount? (reference amount:1 slice 70g salted cod, salted Hokke, or salted salmon)	Eating habits	0.00
How often do you consume powdered green tea?	Eating habits	0.00
Is your daily food intake in the past year higher than the reference amount? (reference amount: 1/2 of a peach, 65g)	Eating habits	0.00
Is your daily food intake in the past year higher than the reference amount? (reference amount: 2 cookies, 25g)	Eating habits	0.00
During this pregnancy, did you handle permanent marker at work at least once a month?	Working condition	0.00

Do you add suger to your tea?	Eating habits	0.00	Do you add suger to your coffee?	Eating habits	0.00
How often do you consume soy milk?	Eating habits	0.00	Occupation: unemployed	Occupation	0.00
Are you living with your partner?	Household composition	0.00	How often do you consume udon?	Eating habits	0.00
Is your daily food intake in the past year higher than the reference amount? (reference amount:1 daikon radish, cut into 2cm slices 80g)	Eating habits	0.00	How often do you consume fried chicken?	Eating habits	0.00
Is your daily food intake in the past year higher than the reference amount? (reference amount:1/2 slice of cod or karei (40g))	Eating habits	0.00	Occupation: administrative public official	Occupation	0.00
How often do you consume fried chicken?	Eating habits	0.00	Is your daily food intake in the past year higher than the reference amount? (reference amount:1 daikon radish, cut into 2cm slices 80g)	Eating habits	0.00
Current employment status	Occupation	0.00	Diagnosis received during past pregnancy: hydatidiform mole	Disease history	0.00
Occupation: sales position	Occupation	0.00	How often do you consume simmered chicken?	Eating habits	0.00
Occupation: social welfare specialists	Occupation	0.00	Occupation: production Work	Occupation	0.00
Is your daily food intake in the past year higher than the reference amount? (reference amount: about 150g per steak)	Eating habits	0.00	Diagnosis received during past pregnancy: ectopic pregnancy	Disease history	0.00
Have you had the flu in the past 6 months?	Disease history	0.00	Disease History: adenomyosis	Disease history	0.00
How often do you consume seaweed and kelp?	Eating habits	0.00	Occupation: police officers and other judicial police personnel	Occupation	0.00
How often do you consume grape?	Eating habits	0.00	Diagnosis received during past pregnancy: Gestational Diabetes Mellitus	Disease history	0.00
Time spent playing games per day (hours)	Physical activity	0.00	How often do you consume fried tofu?	Eating habits	0.00
How often do you consume deep-fried tofu?	Eating habits	0.00	Is your daily food intake in the past year higher than the reference amount? (reference amount: 1 piece of salmon or trout fillet, 70g)	Eating habits	0.00
Is your daily food intake in the past year higher than the reference amount? (reference amount:1 small dish of pickled vegetables (eggplant)(30 g))	Eating habits	0.00	Is your daily food intake in the past year higher than the reference amount? (reference amount: 15g per piece of loin ham)	Eating habits	0.00
Do you add suger to your coffee?	Eating habits	0.00	Reason for changing eating habits: other	Eating habits	0.00
How often do you consume chicken liver?	Eating habits	0.00	Is your daily food intake in the past year higher than the reference amount? (reference amount: 5 tofu cubes, 20g)	Eating habits	0.00
Disease History: dysmenorrhea	Disease history	0.00	Is your daily food intake in the past year higher than the reference amount? (reference amount: 2g of deep-fried tofu for 1 cup of miso soup)	Eating habits	0.00
How often do you consume potato?	Eating habits	0.00	How often do you consume sauteed chicken?	Eating habits	0.00
How often do you consume peanuts and peanuts?	Eating habits	0.00	During this pregnancy, did you handle organic solvent at work at least once a month?	Working condition	0.00
Is your daily food intake in the past year higher than the reference amount? (reference amount: 5 tofu cubes, 20g)	Eating habits	0.00	Is your daily food intake in the past year higher than the reference amount? (reference amount: 2 heads of spinach, 50g)	Eating habits	0.00
Is your daily food intake in the past year higher than the reference amount? (reference amount: 20 peanuts, 20g)	Eating habits	0.00	How often do you consume taro?	Eating habits	0.00
How often do you eating out?	Eating habits	0.00	Time spent playing games per day (hours)	Physical activity	0.00
Disease History: Other obstetric and gynecological diseases	Disease history	0.00	Is your daily food intake in the past year higher than the reference amount? (reference amount: 1 teaspoon ketchup, 5g)	Eating habits	0.00
Are you living with your siblings?	Household composition	0.00	Is your daily food intake in the past year higher than the reference amount? (reference amount: 2 slices of fish paste, 20g)	Eating habits	0.00
Reason for changing eating habits: other	Eating habits	0.00	Current employment status	Occupation	0.00
Is your daily food intake in the past year higher than the reference amount? (reference amount:1 slice of bread, 6 slices(60 g))	Eating habits	0.00	How often do you consume canned coffee?	Eating habits	0.00
How often do you consume sweet potato?	Eating habits	0.00	How often do you consume apple juice?	Eating habits	0.00
How often do you consume pumpkin?	Eating habits	0.00	How often do you consume pickles (umeboshi)?	Eating habits	0.00

How often do you consume chinese cabbage?	Eating habits	0.00
How often do you consume canned coffee?	Eating habits	0.00
Occupation: product sales	Occupation	0.00
How often do you consume apple juice?	Eating habits	0.00
During this pregnancy, did you handle a copier or laser printer at work at least once a month?	Working condition	0.00
How often do you consume cheese?	Eating habits	0.00
How often do you consume spinach?	Eating habits	0.00
How often do you consume wieners and sausages?	Eating habits	0.00
How often do you consume shiitake mushroom?	Eating habits	0.00
How often do you consume broccoli?	Eating habits	0.00
How many cups of miso soup do you drink a day?	Eating habits	0.00
How long does it take to fall asleep?	Sleeping condition	0.00
How often do you consume melon?	Eating habits	0.00
How often do you consume mayonnaise?	Eating habits	0.00
How often do you consume sauteed chicken?	Eating habits	0.00
How often do you consume green bell pepper?	Eating habits	0.00
How often do you consume margarine on bread?	Eating habits	0.00
How often do you consume peach?	Eating habits	0.00
How often do you consume salmon/trout?	Eating habits	0.00
AIS score	Sleeping condition	0.00
Is your daily food intake in the past year higher than the reference amount? (reference amount: 5 thin slices of grilled meat, 100g)	Eating habits	0.00
How often do you consume cookies?	Eating habits	0.00
How often do you consume fried tofu?	Eating habits	0.00
How often do you consume eggplant?	Eating habits	0.00
How often do you consume strawberry?	Eating habits	0.00
Do you wake up in the middle of the night in the middle of your sleep?	Sleeping condition	0.00
How often do you consume tap water and well water?	Eating habits	0.00
How often do you consume vinaigrette?	Eating habits	0.00
How often do you consume cucumber?	Eating habits	0.00
How often do you consume dried laver?	Eating habits	0.00
Total sleeping hours	Sleeping condition	0.00
Is your daily food intake in the past year higher than the reference amount? (reference amount: 1 eggplant, 60g)	Eating habits	0.00
How often do you consume tea?	Eating habits	0.00
Is your daily food intake in the past year higher than the reference amount? (reference amount: 3 slices of squid, 50g)	Eating habits	0.00
Is your daily food intake in the past year higher than the reference amount? (reference amount:1/6 sweet potatoes (40g))	Eating habits	0.00
How often do you consume bacon?	Eating habits	0.00
Occupation: product inspection (metal products)	Occupation	0.00
Occupation: transportation and postal work	Occupation	0.00
Are you living with your child?	Household composition	0.00
How often do you consume melon?	Eating habits	0.00
How often do you consume cookies?	Eating habits	0.00
Is your daily food intake in the past year higher than the reference amount? (reference amount: 2 mandarins, 140g)	Eating habits	0.00
Occupation: accounting clerk	Occupation	0.00
Occupation: production Process Workers	Occupation	0.00
Are you living alone?	Household composition	0.00
Is your daily food intake in the past year higher than the reference amount? (reference amount: 1/2 skewer of eel, 50g)	Eating habits	0.00
How often do you consume peanuts and peanuts?	Eating habits	0.00
How often do you consume cheese?	Eating habits	0.00
Occupation: sales and sales administration	Occupation	0.00
How did you feel when you found out you were pregnant?	Emotional condition	0.00
Occupation: construction worker	Occupation	0.00
Occupation: medical technician	Occupation	0.00
Is your daily food intake in the past year higher than the reference amount? (reference amount:3 thin slices of stir-fried pork(60g))	Eating habits	0.00
Is your daily food intake in the past year higher than the reference amount? (reference amount:1 komatsuna plant, 20g)	Eating habits	0.00
Fertility Treatment: Artificial Fertilization	Feartility treatment	0.00
Is your daily food intake in the past year higher than the reference amount? (reference amount: 2 pieces of konnyaku/shirataki oden, 50g)	Eating habits	0.00
How often do you consume cabbage?	Eating habits	0.00
During the last 30 days, about how often did you feel so depressed that nothing could cheer you up?	K6	0.00
How often do you consume salted dod/salted hokke/salted salmon?	Eating habits	0.00
Are you living with your siblings?	Household composition	0.00
Is your daily food intake in the past year higher than the reference amount? (reference amount:3 thin slices of stir-fried beef(60g))	Eating habits	0.00
How often do you consume margarine on bread?	Eating habits	0.00

Frequency of drinking	Alcohol consumption	0.00
Did you handle kerosene, petroleum, benzene and gasoline at work at least once a month during this pregnancy?	Working condition	0.00
How often do you consume egg?	Eating habits	0.00
How often do you consume bacon?	Eating habits	0.00
Did you ever have to carry anything heavier than about 10 kg?	Working condition	0.00
How often do you consume garlic chive?	Eating habits	0.00
Do you have metal in your teeth?	Dental health	0.00
How often do you consume lettuce?	Eating habits	0.00
How often do you consume pickles (Hakusai)?	Eating habits	0.00
How many days a week do you work?	Working condition	0.00
How often do you consume soba?	Eating habits	0.00
How often do you consume daikon?	Eating habits	0.00
How often do you consume pickles (umeboshi)?	Eating habits	0.00
How often do you consume shrimp?	Eating habits	0.00
How often do you consume salted dod/salted hokke/salted salmon?	Eating habits	0.00
How often do you consume cabbage?	Eating habits	0.00
How often do you consume dried fish?	Eating habits	0.00
How often do you consume bread?	Eating habits	0.00
How often do you consume pickles (cucumber)?	Eating habits	0.00
How often do you consume yakitori?	Eating habits	0.00
How often do you consume apple pear?	Eating habits	0.00
Are you living with your father?	Household composition	0.00
When to start taking folic acid	Folic acid taking	0.00
Time spent watching TV per day (hours)	Physical activity	0.00
Is your daily food intake in the past year higher than the reference amount? (reference amount: chicken liver, 30g per grilled chicken)	Eating habits	0.00
Time spent lying down per day	Physical activity	0.00
Did you use any medications or supplements between 1 year before and when you found out you were pregnant?	Working condition	0.00
Symptoms of Morning Sickness	Body condition	0.00
Duration of passive smoking by junior high school students (years)	Smoking habits	0.00
How often do you consume fried beef?	Eating habits	0.00
How often do you consume rice cake?	Eating habits	0.00
How often do you consume roast ham?	Eating habits	0.00
How often do you consume jam and marmalade for bread?	Eating habits	0.00
Number of days in a week that you walked for at least 10 minutes	Physical activity	0.00
How often do you consume jam and marmalade for bread?	Eating habits	0.00
How often do you consume bonito/tuna	Eating habits	0.00
Time spent lying down per day	Physical activity	0.00
How often do you consume ramen?	Eating habits	0.00
Passive smoking in the year before pregnancy hours per day	Smoking habits	0.00
How often do you consume mayonnaise?	Eating habits	0.00
Number of smokers in the room where you live	Smoking habits	0.00
How often do you consume apple?	Eating habits	0.00
Occupation: security profession worker	Occupation	0.00
How often do you consume broccoli?	Eating habits	0.00
How often do you consume garlic chive?	Eating habits	0.00
How often do you consume deep-fried tofu?	Eating habits	0.00
Do you have metal in your teeth?	Dental health	0.00
How often do you consume tofu (boiled tofu, chilled tofu)?	Eating habits	0.00
During this pregnancy, did you handle a copier or laser printer at work at least once a month?	Working condition	0.00
Is your daily food intake in the past year higher than the reference amount? (reference amount:1/4 of cod roe(25g))	Eating habits	0.00
Occupation: nursing-care service profession	Occupation	0.00
Do you drink alcohol?	Alcohol consumption	0.00
How often do you consume cod roe?	Eating habits	0.00
How often do you consume strawberry?	Eating habits	0.00
Is your daily food intake in the past year higher than the reference amount? (reference amount: 1 small cup of natto, 50g)	Eating habits	0.00
Is your daily food intake in the past year higher than the reference amount? (reference amount: 1/2 sheet of chocolate, 25g)	Eating habits	0.00
Is your daily food intake in the past year higher than the reference amount? (reference amount:1/3 cucumber 30g)	Eating habits	0.00
Did you handle microorganisms at work at least once a month during this pregnancy?	Working condition	0.00
How often do you consume natto?	Eating habits	0.00
Occupation: administrative professional	Occupation	0.00
Is your daily food intake in the past year higher than the reference amount? (reference amount: 250g per bowl of udon)	Eating habits	0.00
Frequency of drinking	Alcohol consumption	0.00
During this pregnancy, did you handle radiation, radioactive materials, or isotopes at work at least once a month?	Working condition	0.00
K6 score	K6	0.00
Is your daily food intake in the past year higher than the reference amount? (reference amount: 5 strawberries, 75g)	Eating habits	0.00
Is your daily food intake in the past year higher than the reference amount? (reference amount:2 pcs Taisho shrimp(40g))	Eating habits	0.00

Do you drink the broth of ramen, udon, or soba noodles?	Eating habits	0.00
How often do you consume Konnyaku and shirataki?	Eating habits	0.00
How often do you consume cod roe?	Eating habits	0.00
How often do you consume carrot?	Eating habits	0.00
How often do you consume taro?	Eating habits	0.00
How often do you consume fat of meat?	Eating habits	0.00
Do you drink alcohol?	Alcohol consumption	0.00
How often do you consume natto?	Eating habits	0.00
Diagnosis received during past pregnancy: threatened preterm labor	Disease history	0.00
How often do you consume canned tuna?	Eating habits	0.00
Do you take naps?	Sleeping condition	0.00
How often do you consume low fat milk?	Eating habits	0.00
During the last 30 days, about how often did you feel worthless?	K6	0.00
Number of days in a week that you walked for at least 10 minutes	Physical activity	0.00
How often do you consume komatsuna?	Eating habits	0.00
How often do you consume soup?	Eating habits	0.00
How often do you consume pickles (eggplant)?	Eating habits	0.00
How often do you consume deep-fried pork?	Eating habits	0.00
How often do you consume powdered green tea?	Eating habits	0.00
Are you satisfied with your overall sleep quality?	Sleeping condition	0.00
Frequency of taking folic acid	Folic acid taking	0.00
How often do you consume mandarin orange?	Eating habits	0.00
Weight before pregnancy	Basic characteristics	0.00
How often do you consume source?	Eating habits	0.00
Occupation: occupations that do not fit into any of the above categories	Occupation	0.00
Number of smokers in the family	Smoking habits	0.00
How often do you consume apple?	Eating habits	0.00
How often do you consume dried young sardines?	Eating habits	0.00
How often do you consume japanese sweets?	Eating habits	0.00
How often do you consume hijiki?	Eating habits	0.00
How often do you consume cake?	Eating habits	0.00
How often do you consume tofu (for miso soup)?	Eating habits	0.00

Occupation: other transportation workers	Occupation	0.00
Symptoms of Morning Sickness	Body condition	0.00
Did you use a machine with strong vibrations at work?	Working condition	0.00
Partner's smoking habit	Smoking habits	0.00
Is your daily food intake in the past year higher than the reference amount? (reference amount:1 small plate of pickles (cucumbers) 30 g))	Eating habits	0.00
During the last 30 days, about how often did you feel nervous?	K6	0.00
Is your daily food intake in the past year higher than the reference amount? (reference amount:1 slice of cheese (20g))	Eating habits	0.00
How often do you consume fat of meat?	Eating habits	0.00
Is your daily food intake in the past year higher than the reference amount? (reference amount:1 slice of bacon 20g)	Eating habits	0.00
Occupation: other office workers	Occupation	0.00
Is your daily food intake in the past year higher than the reference amount? (reference amount: 1/2 apple, 85g)	Eating habits	0.00
Did you handle general anesthetics at work at least once a month during this pregnancy?	Working condition	0.00
Is your daily food intake in the past year higher than the reference amount? (reference amount: 10 large grapes, 100g)	Eating habits	0.00
Do you drink the broth of ramen, udon, or soba noodles?	Eating habits	0.00
Is your daily food intake in the past year higher than the reference amount? (reference amount:1 slice of small shortcake 70g)	Eating habits	0.00
How often do you consume dried laver?	Eating habits	0.00
Is your daily food intake in the past year higher than the reference amount? (reference amount: 1 teaspoon of sauce, 5 g)	Eating habits	0.00
Is your daily food intake in the past year higher than the reference amount? (reference amount: chicken liver, 30g per grilled chicken)	Eating habits	0.00
Occupation: other production process workers	Occupation	0.00
Did you handle mercury at work at least once a month during this pregnancy?	Working condition	0.00
Did you ever have to carry anything heavier than about 10 kg?	Working condition	0.00
How often do you consume pacific saury and mackerel?	Eating habits	0.00
Is your daily food intake in the past year higher than the reference amount? (reference amount:1/4 green onion (20g))	Eating habits	0.00
Occupation: artists, photographers, designers, videographers	Occupation	0.00
How often do you consume cucumber?	Eating habits	0.00
Did you handle medical disinfectant sterilizers at work at least once a month during this pregnancy?	Working condition	0.00
How often do you consume horse mackerel and sardine?	Eating habits	0.00
How often do you consume oyster?	Eating habits	0.00
AIS score	Sleeping condition	0.00
How often do you consume pickles (cucumber)?	Eating habits	0.00
Is your daily food intake in the past year higher than the reference amount? (reference amount: 1/8 of a pineapple, 130g)	Eating habits	0.00
How often do you consume tomato?	Eating habits	0.00

How often do you consume udon?	Eating habits	0.00
How often do you consume shimeji?	Eating habits	0.00
How often do you consume onion?	Eating habits	0.00
How often do you consume fried food?	Eating habits	0.00
What is the most frequent way to cook vegitables?	Eating habits	0.00
How often do you consume pacific saury and mackerel?	Eating habits	0.00
How often do you consume asari and shijimi?	Eating habits	0.00
How often do you consume instant foods?	Eating habits	0.00
What type of oil do you use most often?	Eating habits	0.00
How often do you consume tea?	Eating habits	0.00
How often do you consume bonito/tuna	Eating habits	0.00
Is your daily food intake in the past year higher than the reference amount? (reference amount:Pickle (dried plum) in 1 piece (8g))	Eating habits	0.00
How often do you consume oyster?	Eating habits	0.00
How often do you consume fried pork?	Eating habits	0.00
How often do you consume burdock root?	Eating habits	0.00
How often do you consume potable water?	Eating habits	0.00
Number of smokers in the room where you live	Smoking habits	0.00
How often do you consume squid?	Eating habits	0.00
How did you feel when you found out you were pregnant?	Emotional condition	0.00
How often do you consume tomato?	Eating habits	0.00
How often do you consume yellowtail amberjack?	Eating habits	0.00
During an average week before pregnancy, were there any days of intense physical activity?	Physical activity	0.00
Partner's smoking habit	Smoking habits	0.00
How often do you consume simmered pork?	Eating habits	0.00
How often do you consume tube-shaped fish-paste cake?	Eating habits	0.00
How often do you consume chocolate?	Eating habits	0.00
How often do you consume eel?	Eating habits	0.00
How often do you consume pork liver?	Eating habits	0.00
How often do you consume tofu (boiled tofu, chilled tofu)?	Eating habits	0.00
How often do you consume fermented milk drink?	Eating habits	0.00
How often do you consume ketchup?	Eating habits	0.00
How often do you consume stewed Beef?	Eating habits	0.00
How often do you consume pork soup?	Eating habits	0.00

How often do you consume shrimp?	Eating habits	0.00
How often do you consume shimeji?	Eating habits	0.00
Occupation: sales-related position	Occupation	0.00
Is your daily food intake in the past year higher than the reference amount? (reference amount:3 cubes of stewed beef(50g))	Eating habits	0.00
Occupation: other professional occupations	Occupation	0.00
Is your daily food intake in the past year higher than the reference amount? (reference amount: 50g chicken cubes)	Eating habits	0.00
When to start taking folic acid	Folic acid taking	0.00
Number of smokers in the family	Smoking habits	0.00
How often do you consume squid?	Eating habits	0.00
How often do you consume dried fish?	Eating habits	0.00
How often do you consume tofu (for miso soup)?	Eating habits	0.00
How often do you consume tap water and well water?	Eating habits	0.00
Do you take naps?	Sleeping condition	0.00
How often do you consume spinach?	Eating habits	0.00
What is the most frequent way to cook seafood?	Eating habits	0.00
Is your daily food intake in the past year higher than the reference amount? (reference amount: 220g per bowl of ramen)	Eating habits	0.00
How often do you consume fermented milk drink?	Eating habits	0.00
Occupation: transport and machine operator (transport)	Occupation	0.00
Occupation: manufacturing engineers (excluding development)	Occupation	0.00
Occupation: Packaging Worker	Occupation	0.00
Did you handle cancer drugs at work at least once a month during this pregnancy?	Working condition	0.00
Did you handle formalin and formaldehyde at work at least once a month during this pregnancy?	Working condition	0.00
How often do you consume potato?	Eating habits	0.00
Is your daily food intake in the past year higher than the reference amount? (reference amount:1/4 carrot (50g))	Eating habits	0.00
How often do you consume potable water?	Eating habits	0.00
Occupation: health and Medical Services	Occupation	0.00
Is your daily food intake in the past year higher than the reference amount? (reference amount:1/2 slice of cod or karei (40g))	Eating habits	0.00
Occupation: customer service and catering	Occupation	0.00
Occupation: Administrative staff of corporations, organizations	Occupation	0.00
Diagnosis received during past pregnancy: Placenta praevia	Disease history	0.00
Is your daily food intake in the past year higher than the reference amount? (reference amount: 1 taro, 30g)	Eating habits	0.00
Did you handle other chemicals at work at least once a month during this pregnancy?	Working condition	0.00
Is your daily food intake in the past year higher than the reference amount? (reference amount:Pickle (dried plum) in 1 piece (8g))	Eating habits	0.00

Is your daily food intake in the past year higher than the reference amount? (reference amount: 1/2 sheet of chocolate, 25g)	Eating habits	0.00	How often do you consume stewed Beef?	Eating habits	0.00
How often do you consume horse mackerel and sardine?	Eating habits	0.00	How often do you consume ketchup?	Eating habits	0.00
Is your daily food intake in the past year higher than the reference amount? (reference amount:1 slice 70g salted cod, salted Hokke, or salted salmon)	Eating habits	0.00	How often do you consume cake?	Eating habits	0.00
Occupation: customer service and catering	Occupation	0.00	How often do you consume eggplant?	Eating habits	0.00
Is your daily food intake in the past year higher than the reference amount? (reference amount:1 banana (75g))	Eating habits	0.00	How often do you consume seaweed and kelp?	Eating habits	0.00
Have you ever had to work between the hours of 10 p.m. and dawn?	Working condition	0.00	How often do you consume tube-shaped fish-paste cake?	Eating habits	0.00
What is the most frequent way to cook seafood?	Eating habits	0.00	Occupation: fisherman	Occupation	0.00
Is your daily food intake in the past year higher than the reference amount? (reference amount:1 small bowl of hijiki(20g))	Eating habits	0.00	How many days a week do you work?	Working condition	0.00
During this pregnancy, did you handle organic solvent at work at least once a month?	Working condition	0.00	Occupation: cleanup crew	Occupation	0.00
How sleepy are you during the day?	Sleeping condition	0.00	Do you wake up in the middle of the night in the middle of your sleep?	Sleeping condition	0.00
How often do you consume beef steak?	Eating habits	0.00	Is your daily food intake in the past year higher than the reference amount? (reference amount:1/4 tofu 75g)	Eating habits	0.00
Disease History: Ovarian Tumor/Ovarian Cyst	Disease history	0.00	Time spent watching TV per day (hours)	Physical activity	0.00
Is your daily food intake in the past year higher than the reference amount? (reference amount: 2 pieces of konnyaku/shirataki oden, 50g)	Eating habits	0.00	How often do you consume yakitori?	Eating habits	0.00
Occupation: production Work	Occupation	0.00	Did you handle herbicide at work at least once a month during this pregnancy?	Working condition	0.00
Occupation: unemployed	Occupation	0.00	Is your daily food intake in the past year higher than the reference amount? (reference amount: 1/2 tbsp mayonnaise, 7g)	Eating habits	0.00
How often do you consume octopus?	Eating habits	0.00	Diagnosis received during past pregnancy: Twin pregnancy	Disease history	0.00
Is your daily food intake in the past year higher than the reference amount? (reference amount: 200g buckwheat noodles per bowl)	Eating habits	0.00	How often do you consume lettuce?	Eating habits	0.00
How often do you consume papaya?	Eating habits	0.00	During an average week before pregnancy, were there any days of intense physical activity?	Physical activity	0.00
Size of the serving bowl	Eating habits	0.00	How often do you consume papaya?	Eating habits	0.00
Occupation: faculty	Occupation	0.00	Did you use any medications or supplements between 1 year before and when you found out you were pregnant?	Working condition	0.00
Is your daily food intake in the past year higher than the reference amount? (reference amount: 1 medium egg, 50g)	Eating habits	0.00	Occupation: agricultural, forestry, and fishery workers	Occupation	0.00
Did you handle medical disinfectant sterilizers at work at least once a month during this pregnancy?	Working condition	0.00	How often do you consume burdock root?	Eating habits	0.00
Is your daily food intake in the past year higher than the reference amount? (reference amount:(1 yogurt cup mold(120 g))	Eating habits	0.00	How often do you consume simmered pork?	Eating habits	0.00
Reason for changing eating habits: thought it was good for my health	Eating habits	0.00	How many cups of miso soup do you drink a day?	Eating habits	0.00
Occupation: other office workers	Occupation	0.00	How often do you consume green bell pepper?	Eating habits	0.00
Is your daily food intake in the past year higher than the reference amount? (reference amount: 250g per bowl of udon)	Eating habits	0.00	How often do you consume eel?	Eating habits	0.00
Is your daily food intake in the past year higher than the reference amount? (reference amount: 2g of deep-fried tofu for 1 cup of miso soup)	Eating habits	0.00	How often do you consume fried food?	Eating habits	0.00
Is your daily food intake in the past year higher than the reference amount? (reference amount: 220g per bowl of ramen)	Eating habits	0.00	Duration of passive smoking by junior high school students (years)	Smoking habits	0.00
Diagnosis received during past pregnancy: ectopic pregnancy	Disease history	0.00	Frequency of taking folic acid	Folic acid taking	0.00

Diagnosis received during past pregnancy: Gestational Diabetes Mellitus	Disease history	0.00
Diagnosis received during past pregnancy: hydatidiform mole	Disease history	0.00
Diagnosis received during past pregnancy: Other obstetric and gynecological diseases	Disease history	0.00
Diagnosis received during past pregnancy: placenta abruption	Disease history	0.00
Diagnosis received during past pregnancy: Placenta praevia	Disease history	0.00
Diagnosis received during past pregnancy: Twin pregnancy	Disease history	0.00
Disease History: adenomyosis	Disease history	0.00
Disease History: malformation of the uterus	Disease history	0.00
Have you had mumps in the past 6 months?	Disease history	0.00
Have you had rubella in the past six months?	Disease history	0.00
Reason for change in eating habits: Abnormalities in tests	Eating habits	0.00
Reason for change in eating habits: Change in taste	Eating habits	0.00
Reason for change in eating habits: got sick	Eating habits	0.00
Are you living alone?	Household composition	0.00
Fertility Treatment: Artificial Fertilization	Feartility treatment	0.00
Fertility Treatment: Other	Feartility treatment	0.00
Occupation: accounting clerk	Occupation	0.00
Occupation: administrative professional	Occupation	0.00
Occupation: administrative public official	Occupation	0.00
Occupation: Administrative staff of corporations, organizations	Occupation	0.00
Occupation: agricultural, forestry, and fishery workers	Occupation	0.00
Occupation: artists, photographers, designers, videographers	Occupation	0.00
Occupation: author, reporter, editor	Occupation	0.00
Occupation: carrier	Occupation	0.00
Occupation: cleanup crew	Occupation	0.00
Occupation: construction worker	Occupation	0.00
Occupation: farmer	Occupation	0.00
Occupation: fisherman	Occupation	0.00
Occupation: general office worker	Occupation	0.00
Occupation: health and Medical Services	Occupation	0.00
Occupation: information processing and telecommunications rngineer	Occupation	0.00
Occupation: management, finance and insurance profession	Occupation	0.00
Occupation: manufacturing engineers (excluding development)	Occupation	0.00
Occupation: medical technician	Occupation	0.00
Occupation: nursing-care service profession	Occupation	0.00
Occupation: Officers of corporations and organizations	Occupation	0.00

How often do you consume shiitake mushroom?	Eating habits	0.00
Occupation: management, finance and insurance profession	Occupation	0.00
What type of oil do you use most often?	Eating habits	0.00
How often do you consume mandarin orange?	Eating habits	0.00
How often do you consume wieners and sausages?	Eating habits	0.00
Is your daily food intake in the past year higher than the reference amount? (reference amount:1/4 canned tuna 20g)	Eating habits	0.00
How often do you consume pork liver?	Eating habits	0.00
How often do you consume dried young sardines?	Eating habits	0.00
How often do you consume chicken liver?	Eating habits	0.00
Occupation: other administrative staf	Occupation	0.00
How often do you consume hijiki?	Eating habits	0.00
How often do you consume komatsuna?	Eating habits	0.00
Size of the serving bowl	Eating habits	0.00
Is your daily food intake in the past year higher than the reference amount? (reference amount: 1/4 of a shimeji mushroom, 20g)	Eating habits	0.00
Fertility Treatment: Other	Feartility treatment	0.00
How often do you consume salmon/trout?	Eating habits	0.00
Occupation: general office worker	Occupation	0.00
Occupation: Officers of corporations and organizations	Occupation	0.00
Occupation: faculty	Occupation	0.00
How often do you consume fried beef?	Eating habits	0.00
Occupation: other service occupations	Occupation	0.00
Is your daily food intake in the past year higher than the reference amount? (reference amount:1/8 watermelon 120g)	Eating habits	0.00
Have you ever had to work between the hours of 10 p.m. and dawn?	Working condition	0.00
How often do you consume pickles (eggplant)?	Eating habits	0.00
How often do you consume japanese sweets?	Eating habits	0.00
How often do you consume Konnyaku and shirataki?	Eating habits	0.00
Occupation: production-related office work	Occupation	0.00
What is the most frequent way to cook vegitables?	Eating habits	0.00
How often do you consume onion?	Eating habits	0.00
How often do you consume octopus?	Eating habits	0.00
How often do you consume pickles (Hakusai)?	Eating habits	0.00
How often do you consume vinaigrette?	Eating habits	0.00
Did you handle solder or other lead-containing products at work at least once a month during this pregnancy?	Working condition	0.00
How often do you consume pumpkin?	Eating habits	0.00
Is your daily food intake in the past year higher than the reference amount? (reference amount: 5 thin slices of grilled meat, 100g)	Eating habits	0.00
During this pregnancy, did you handle engine oil at work at least once a month?	Working condition	0.00

Occupation: other administrative staf	Occupation	0.00
Occupation: other health care professionals	Occupation	0.00
Occupation: other production process workers	Occupation	0.00
Occupation: other professional occupations	Occupation	0.00
Occupation: other service occupations	Occupation	0.00
Occupation: other technicians	Occupation	0.00
Occupation: other transportation workers	Occupation	0.00
Occupation: Packaging Worker	Occupation	0.00
Occupation: police officers and other judicial police personnel	Occupation	0.00
Occupation: product inspection (metal products)	Occupation	0.00
Occupation: production Process Workers	Occupation	0.00
Occupation: production-related office work	Occupation	0.00
Occupation: researcher	Occupation	0.00
Occupation: sales and sales administration	Occupation	0.00
Occupation: sales-related position	Occupation	0.00
Occupation: security profession worker	Occupation	0.00
Occupation: self-defense official	Occupation	0.00
Occupation: transport and machine operator (transport)	Occupation	0.00
Occupation: transportation and postal work	Occupation	0.00
Have you taken sleeping pills more than three times a week in the past month?	Sleeping condition	0.00
Did you handle cancer drugs at work at least once a month during this pregnancy?	Working condition	0.00
Did you handle formalin and formaldehyde at work at least once a month during this pregnancy?	Working condition	0.00
Did you handle general anesthetics at work at least once a month during this pregnancy?	Working condition	0.00
Did you handle herbicide at work at least once a month during this pregnancy?	Working condition	0.00
Did you handle mercury at work at least once a month during this pregnancy?	Working condition	0.00
Did you handle microorganisms at work at least once a month during this pregnancy?	Working condition	0.00
Did you handle other chemicals at work at least once a month during this pregnancy?	Working condition	0.00
Did you handle pesticides other than those listed above or whose type is not known at work at least once a month during this pregnancy?	Working condition	0.00
Did you handle solder or other lead-containing products at work at least once a month during this pregnancy?	Working condition	0.00
Did you use a machine with strong vibrations at work?	Working condition	0.00

How often do you consume fried pork?	Eating habits	0.00
How often do you consume daikon?	Eating habits	0.00
How often do you consume chocolate?	Eating habits	0.00
How often do you consume instant foods?	Eating habits	0.00
Is your daily food intake in the past year higher than the reference amount? (reference amount:1 small bowl of hijiki(20g))	Eating habits	0.00
Is your daily food intake in the past year higher than the reference amount? (reference amount: 200g buckwheat noodles per bowl)	Eating habits	0.00
How often do you consume beef steak?	Eating habits	0.00
How often do you consume carrot?	Eating habits	0.00
How often do you consume canned tuna?	Eating habits	0.00
Occupation: carrier	Occupation	0.00
How many people, including yourself, live in the house?	Household composition	0.00
How often do you eating out?	Eating habits	0.00
Are you satisfied with your overall sleep quality?	Sleeping condition	0.00
Occupation: author, reporter, editor	Occupation	0.00
Is your daily food intake in the past year higher than the reference amount? (reference amount:(3 cubes of stewed pork(50g))	Eating habits	0.00
Is your daily food intake in the past year higher than the reference amount? (reference amount:1 small bowl of wakame seaweed/kelp 20g)	Eating habits	0.00
Occupation: self-defense official	Occupation	0.00
Is your daily food intake in the past year higher than the reference amount? (reference amount: 1/2 of other citrus fruits, 75g)	Eating habits	0.00
How often do you consume chinese cabbage?	Eating habits	0.00
How often do you consume rice cake?	Eating habits	0.00
Did you handle pesticides other than those listed above or whose type is not known at work at least once a month during this pregnancy?	Working condition	0.00
How often do you consume deep-fried pork?	Eating habits	0.00
How often do you consume egg?	Eating habits	0.00
Is your daily food intake in the past year higher than the reference amount? (reference amount:(1 yogurt cup mold(120 g))	Eating habits	0.00
How often do you consume bread?	Eating habits	0.00
Occupation: farmer	Occupation	0.00
How sleepy are you during the day?	Sleeping condition	0.00
Did you handle kerosene, petroleum, benzene and gasoline at work at least once a month during this pregnancy?	Working condition	0.00
How often do you consume low fat milk?	Eating habits	0.00
How often do you consume soup?	Eating habits	0.00

During this pregnancy, did you handle engine oil at work at least once a month?	Working condition	0.00
During this pregnancy, did you handle radiation, radioactive materials, or isotopes at work at least once a month?	Working condition	0.00

Weight before pregnancy	Basic characteristics	0.00
How often do you consume asari and shijimi?	Eating habits	0.00

GH-(SPE/PE) model

Questionnaires completed in the early stage of pregnancy, RFE, LR, AUC=0.59		
Feature name	Category	Importance score
Total sleeping hours	Sleeping condition	0.04
Are you satisfied with your overall sleep quality?	Sleeping condition	0.04
How often do you consume cookies?	Eating habits	0.04
Occupation: accounting clerk	Occupation	0.03
How often do you consume green onion?	Eating habits	0.03
Is your daily food intake in the past year higher than the reference amount? (reference amount:1 small plate of pickled vegetables (chinese cabbege (30 g))	Eating habits	0.03
How often do you consume bean sprouts?	Eating habits	0.03
Is your daily food intake in the past year higher than the reference amount? (reference amount: 2 thin slices of pork in soup 40g)	Eating habits	0.03
Frequency of consumption of miso soup	Eating habits	0.03
Is your daily food intake in the past year higher than the reference amount? (reference amount:1 slice of bacon 20g)	Eating habits	0.03
What is the most frequent way to cook meat?	Eating habits	0.03
Occupation: nursing-care service profession	Occupation	0.03
Duration of passive smoking by junior high school students (years)	Smoking habits	0.03
How often do you consume fried food?	Eating habits	0.03
Is your daily food intake in the past year higher than the reference amount? (reference amount: 8g margarine for 1 slice of bread)	Eating habits	0.03
Is your daily food intake in the past year higher than the reference amount? (reference amount:1/2 piece of fresh/thickly fried tofu 60g)	Eating habits	0.03
How often do you consume roast ham?	Eating habits	0.03
Height of body	Basic characteristics	0.03
How often do you consume pacific saury and mackerel?	Eating habits	0.03
Is your daily food intake in the past year higher than the reference amount? (reference amount: 8g butter for 1 slice of bread)	Eating habits	0.03
How often do you consume cake?	Eating habits	0.03
How often do you consume burdock root?	Eating habits	0.03
How often do you consume squid?	Eating habits	0.02
How often do you consume pickles (umeboshi)?	Eating habits	0.02
Is your daily food intake in the past year higher than the reference amount? (reference amount: 2 heads of spinach, 50g)	Eating habits	0.02
Disease history : polycystic ovary syndrome	Disease history	0.02
During the last 30 days, about how often did you feel restless or fidgety?	K6	0.02

Laboratory test data collected in the early stage of pregnancy,RFE, LR, AUC=0.58		
Feature name	Category	Importance score
Albumin quantitative value	Renal function	0.35

Is your daily food intake in the past year higher than the reference amount? (reference amount: 2 wiener sausages, 30g)	Eating habits	0.02
Is your daily food intake in the past year higher than the reference amount? (reference amount:(1 yogurt cup mold(120 g))	Eating habits	0.02
How often do you consume canned tuna?	Eating habits	0.02
How often do you consume pickles (eggplant)?	Eating habits	0.02
How often do you consume apple pear?	Eating habits	0.02
How often do you consume grape?	Eating habits	0.02
Is your daily food intake in the past year higher than the reference amount? (reference amount: 1 teaspoon ketchup, 5g)	Eating habits	0.02
How often do you consume pickles (cucumber)?	Eating habits	0.02
Occupation: sales and sales administration	Occupation	0.02
Do you add suger to your tea?	Eating habits	0.02
During the last 30 days, about how often did you feel nervous?	K6	0.01

SPE-PE model

Questionnaires completed in the early stage of pregnancy, RFE, LR, AUC=0.71		
Feature name	Category	Importance score
Height of body	Basic characteristics	0.02
Number of days in a week that you walked for at least 10 minutes	Physical activity	0.02
How often do you consume soft drink?	Eating habits	0.01
Number of children you have living with you	Family living arrangements	0.01
How often do you consume energy drink?	Eating habits	0.01
How often do you consume rice cake?	Eating habits	0.01
How often do you consume bean sprouts?	Eating habits	0.01
Have you taken sleeping pills more than three times a week in the past month?	Sleeping condition	0.01
How often do you consume soba?	Eating habits	0.01
How often do you consume cabbage?	Eating habits	0.01
How often do you consume vinaigrette?	Eating habits	0.01
How often do you consume pork soup?	Eating habits	0.01
Is your daily food intake in the past year higher than the reference amount? (reference amount: 200g buckwheat noodles per bowl)	Eating habits	0.01
How often do you consume green onion?	Eating habits	0.01
Reason for change in eating habits: Abnormalities in tests	Eating habits	0.01
AIS score	Sleeping condition	0.01
How often do you consume garlic chive?	Eating habits	0.01
Occupation: other service occupations	Occupation	0.01
How often do you consume roast ham?	Eating habits	0.01
Is your daily food intake in the past year higher than the reference amount? (reference amount:1/4 bag bean sprouts 25g)	Eating habits	0.01

Laboratory test data collected in the early stage of pregnancy, RFE, RF, AUC=0.70		
Feature name	Category	Importance score
Antibody concentration (Japanese cedar)	Allegy test	0.14
Allegy testing class: mite	Allegy test	0.14
Non-specific IgE	Allegy test	0.10
Antibody concentration (mite)	Allegy test	0.08
γ-GTP	Hepatic function test	0.05
HDL-cholesterol	Lipid profile	0.05
Neutral fat	Lipid profile	0.04
GPT	Hepatic function test	0.04
Albumin quantitative value	Renal function test	0.04
Albumin creatinine ratio	Renal function test	0.04
GFR class	Renal function test	0.03
GOT	Hepatic function test	0.03
Total cholesterol	Lipid profile	0.03
Uric potassium	Urinal test	0.03
Uric Cl	Urinal test	0.03
Urea nitrogen	Renal function test	0.03
Urine sodium	Urinal test	0.03
Uric acid	Blood test	0.02
Urine sodium(for conversion)	Urinal test	0.02
Allegy testing class: cat	Allegy test	0.02

Is your daily food intake in the past year higher than the reference amount? (reference amount: 1/4 burdock root, 40g)	Eating habits	0.01
Reason for changing eating habits: thought it was good for my health	Eating habits	0.01
Occupation: cleanup crew	Occupation	0.01
How often do you consume broccoli?	Eating habits	0.01
During the last 30 days, about how often did you feel worthless?	K6	0.01
Occupation: management, finance and insurance profession	Occupation	0.01
How often do you consume ketchup?	Eating habits	0.01
How often do you consume yakitori?	Eating habits	0.01
Reason for change in eating habits: Change in taste	Eating habits	0.01
Is your daily food intake in the past year higher than the reference amount? (reference amount: 1 piece of salmon or trout fillet, 70g)	Eating habits	0.01
How often do you consume peanuts and peanuts?	Eating habits	0.01
Is your daily food intake in the past year higher than the reference amount? (reference amount:1/4 of cod roe(25g))	Eating habits	0.01
How often do you consume egg?	Eating habits	0.01
Time spent playing games per day (hours)	Physical activity	0.01
How often do you consume wieners and sausages?	Eating habits	0.01
Occupation: production Work	Occupation	0.01
Occupation: construction worker	Occupation	0.01
Is your daily food intake in the past year higher than the reference amount? (reference amount:1/4 tofu 75g)	Eating habits	0.01
Disease History: Other obstetric and gynecological diseases	Disease history	0.01
How often do you consume braised pork?	Eating habits	0.01
Are you living with your partner's father?	Family living arrangements	0.01
How often do you consume pickles (takuan)?	Eating habits	0.01
Is your daily food intake in the past year higher than the reference amount? (reference amount:1 small plate of pickled vegetables (chinese cabbege (30 g))	Eating habits	0.01
Occupation: health and Medical Services	Occupation	0.01
Symptoms of Morning Sickness	Body condition	0.01
Is your daily food intake in the past year higher than the reference amount? (reference amount: 1/2 apple, 85g)	Eating habits	0.01
Occupation: sanitation services (barbers, beauticians, etc.)	Occupation	0.01
How often do you consume sea bream?	Eating habits	0.01
Is your daily food intake in the past year higher than the reference amount? (reference amount: 6 pea pods, 30g)	Eating habits	0.01
How often do you consume mandarin orange?	Eating habits	0.01
How often do you consume orange juice?	Eating habits	0.01
During this pregnancy, did you handle a copier or laser printer at work at least once a month?	Working condition	0.01
Occupation: other health care professionals	Occupation	0.01
How often do you consume tofu (for miso soup)?	Eating habits	0.01
Is your daily food intake in the past year higher than the reference amount? (reference amount: 5 tofu cubes, 20g)	Eating habits	0.01
How often do you consume melon?	Eating habits	0.01
How often do you consume pickles (cucumber)?	Eating habits	0.01
HbA1c	Blood test	0.00
Allegy testing class: cat	Allegy test	0.00
Allegy testing class: shrimp	Allegy test	0.00
Cystatin-C	Renal function test	0.00
Blood creatinine	Renal function test	0.00

How often do you consume tomato?	Eating habits	0.01
How often do you consume instant foods?	Eating habits	0.01
How did you feel during the day?	Sleeping condition	0.01
How often do you consume apple juice?	Eating habits	0.01
Time spent lying down per day	Physical activity	0.01
Are you living with your partner's mother?	Family living arrangements	0.01
Are you satisfied with your overall sleep quality?	Sleeping condition	0.01
How often do you consume seaweed and kelp?	Eating habits	0.01
How often do you consume apple?	Eating habits	0.01
Occupation: police officers and other judicial police personnel	Occupation	0.01
Occupation: security profession worker	Occupation	0.01
How often do you consume stewed Beef?	Eating habits	0.01
How often do you consume coffee?	Eating habits	0.01
Is your daily food intake in the past year higher than the reference amount? (reference amount:(3 cubes of stewed pork(50g))	Eating habits	0.01
How often do you consume canned coffee?	Eating habits	0.01
How often do you consume strawberry?	Eating habits	0.01
How often do you consume cucumber?	Eating habits	0.01
Is your daily food intake in the past year higher than the reference amount? (reference amount:1/4 green onion (20g))	Eating habits	0.01
Is your daily food intake in the past year higher than the reference amount? (reference amount: 8g butter for 1 slice of bread)	Eating habits	0.01
During the last 30 days, about how often did you feel nervous?	K6	0.01
How often do you consume pork liver?	Eating habits	0.01
Is your daily food intake in the past year higher than the reference amount? (reference amount: 1 medium leaf of lettuce, 10g)	Eating habits	0.01
How often do you consume jam and marmalade for bread?	Eating habits	0.01
How often do you consume vitamin-fortified rice?	Eating habits	0.01
Do you wake up in the middle of the night in the middle of your sleep?	Sleeping condition	0.01
Do you mix millet with rice?	Eating habits	0.01
How often do you consume kiwi fruit?	Eating habits	0.01
Frequency of taking folic acid	Folic acid taking	0.01
Frequency of drinking	Alcohol consumption	0.00
Is your daily food intake in the past year higher than the reference amount? (reference amount: 1/2 skewer of eel, 50g)	Eating habits	0.00
How often do you consume oolong tea?	Eating habits	0.00
Is your daily food intake in the past year higher than the reference amount? (reference amount: 1/2 kiwifruit, 50g)	Eating habits	0.00
How often do you consume hijiki?	Eating habits	0.00
Diagnosis received during past pregnancy: Twin pregnancy	Disease history	0.00
Is the miso soup highly seasoned?	Eating habits	0.00
Is your daily food intake in the past year higher than the reference amount? (reference amount: 220g per bowl of ramen)	Eating habits	0.00

Is your daily food intake in the past year higher than the reference amount? (reference amount:1 small bowl of hijiki(20g))	Eating habits	0.00
Diagnosis received during past pregnancy: Hypertensive disorder of pregnancy	Disease history	0.00
Occupation: faculty	Occupation	0.00
What is the most frequent way to cook seafood?	Eating habits	0.00
Are you living with your father?	Family living arrangements	0.00
How often do you consume bonito/tuna	Eating habits	0.00
How often do you consume deep-fried tofu?	Eating habits	0.00
How often do you consume pumpkin?	Eating habits	0.00
Occupation: researcher	Occupation	0.00
How often do you consume shrimp?	Eating habits	0.00
What type of steak/yakiniku do you eat most often?	Eating habits	0.00
How many cups of miso soup do you drink a day?	Eating habits	0.00
Is your daily food intake in the past year higher than the reference amount? (reference amount: 3 bunches of broccoli, 30g)	Eating habits	0.00
Do you have the habit of putting soy sauce on your food at the table?	Eating habits	0.00
Is your daily food intake in the past year higher than the reference amount? (reference amount: 1/3 of a leaf of Chinese cabbage, 30g)	Eating habits	0.00
How often do you consume fried tofu?	Eating habits	0.00
Occupation: public health nurses, midwives, nurses	Occupation	0.00
Do you drink the broth of ramen, udon, or soba noodles?	Eating habits	0.00
Did you handle insecticides at work at least once a month during this pregnancy?	Working condition	0.00
Fertility Treatment: Clomid	Feartility treatment	0.00
During an average week before pregnancy, were there any days of intense physical activity?	Physical activity	0.00
Is your daily food intake in the past year higher than the reference amount? (reference amount:3 thin slices of stir-fried pork(60g))	Eating habits	0.00
Is your daily food intake in the past year higher than the reference amount? (reference amount: 10 clams or clam shells, 20g)	Eating habits	0.00
Is your daily food intake in the past year higher than the reference amount? (reference amount: 3 slices of squid, 50g)	Eating habits	0.00
How often do you consume sauteed chicken?	Eating habits	0.00
What type of oil do you use most often?	Eating habits	0.00
Is your daily food intake in the past year higher than the reference amount? (reference amount: 1 eggplant, 60g)	Eating habits	0.00
How often do you consume spinach?	Eating habits	0.00
How often do you consume onion?	Eating habits	0.00
How often do you consume natto?	Eating habits	0.00
Did you handle medical disinfectant sterilizers at work at least once a month during this pregnancy?	Working condition	0.00
How often do you consume enoki mushroom?	Eating habits	0.00
Is your daily food intake in the past year higher than the reference amount? (reference amount: 8g margarine for 1 slice of bread)	Eating habits	0.00
Is your daily food intake in the past year higher than the reference amount? (reference amount : apple pear 1/2 80g)	Eating habits	0.00

Did you handle formalin and formaldehyde at work at least once a month during this pregnancy?	Working condition	0.00
Is your daily food intake in the past year higher than the reference amount? (reference amount:1 green pepper (30g))	Eating habits	0.00
Is your daily food intake in the past year higher than the reference amount? (reference amount: 8g of jam or marmalade per slice of bread)	Eating habits	0.00
Is your daily food intake in the past year higher than the reference amount? (reference amount:1 piece of dried fish, 50g))	Eating habits	0.00
How often do you consume shiitake mushroom?	Eating habits	0.00
Is your daily food intake in the past year higher than the reference amount? (reference amount: 2 wiener sausages, 30g)	Eating habits	0.00
How often do you consume grape?	Eating habits	0.00
Partner's smoking habit	Smoking habits	0.00
Is your daily food intake in the past year higher than the reference amount? (reference amount:3 slices pickles (takuan) 30g)	Eating habits	0.00
Is your daily food intake in the past year higher than the reference amount? (reference amount: 1/2 of other citrus fruits, 75g)	Eating habits	0.00
How often do you consume fried food?	Eating habits	0.00
Have you had the flu in the past 6 months?	Disease history	0.00
Smoking habit	Smoking habits	0.00
During this pregnancy, did you handle permanent marker at work at least once a month?	Working condition	0.00
Do you mix barley with rice?	Eating habits	0.00
How often do you consume carrot?	Eating habits	0.00
Is your daily food intake in the past year higher than the reference amount? (reference amount: 1 shiitake mushroom, 20g)	Eating habits	0.00
How often do you consume deep-fried foods?	Eating habits	0.00
How often do you consume squid?	Eating habits	0.00
How often do you consume butter for bread?	Eating habits	0.00
Is your residence or workplace a noisy environment?	Living condition	0.00
Is your daily food intake in the past year higher than the reference amount? (reference amount: 50g chicken cubes)	Eating habits	0.00
Are your daytime activities the same as usual?	Sleeping condition	0.00
How often do you consume tap water and well water?	Eating habits	0.00
Did you handle water-based paint and inkjet printer at work at least once a month during this pregnancy?	Working condition	0.00
How often do you consume pickles (Hakusai)?	Eating habits	0.00
Is your daily food intake in the past year higher than the reference amount? (reference amount: 1/2 sheet of Koya tofu/frozen bean curd 60g)	Eating habits	0.00
Have you undergone fertility treatment?	Feartility treatment	0.00
How often do you consume brown tea?	Eating habits	0.00
Is your daily food intake in the past year higher than the reference amount? (reference amount: 1/4 of a shimeji mushroom, 20g)	Eating habits	0.00
Occupation : Transportation/cleaning/packaging worker	Occupation	0.00

Is your daily food intake in the past year higher than the reference amount? (reference amount: 1/3 bunch of shunugiku, 30g)	Eating habits	0.00
How often do you consume yellowtail amberjack?	Eating habits	0.00
How often do you consume cheese?	Eating habits	0.00
Current employment status	Occupation	0.00
How often do you consume japanese sweets?	Eating habits	0.00
During this pregnancy, did you handle radiation, radioactive materials, or isotopes at work at least once a month?	Working condition	0.00
Are you taking any supplements that contain folic acid as an ingredient?	Folic acid taking	0.00
Did you handle chlorine bleach and disinfectant at work at least once a month during this pregnancy?	Working condition	0.00
How often do you consume banana?	Eating habits	0.00
Occupation: Officers of corporations and organizations	Occupation	0.00
Occupation: musician, performing artist	Occupation	0.00
Did you handle kerosene, petroleum, benzene and gasoline at work at least once a month during this pregnancy?	Working condition	0.00
Is your daily food intake in the past year higher than the reference amount? (reference amount: 2 tablespoons of shirasu-boshi, 10g)	Eating habits	0.00
Is your daily food intake in the past year higher than the reference amount? (reference amount: 2 slices of boiled pork, 40g)	Eating habits	0.00
Occupation: product sales	Occupation	0.00
How often do you consume oyster?	Eating habits	0.00
Is your daily food intake in the past year higher than the reference amount? (reference amount:1/4 carrot (50g))	Eating habits	0.00
Is your daily food intake in the past year higher than the reference amount? (reference amount: 2 pieces of konnyaku/shirataki oden, 50g)	Eating habits	0.00
Is your daily food intake in the past year higher than the reference amount? (reference amount: 1 tablespoon of dressing, 10g)	Eating habits	0.00
Is your daily food intake in the past year higher than the reference amount? (reference amount: 1/2 oyster, 80 g)	Eating habits	0.00
Occupation: other office workers	Occupation	0.00
Are you living with your partner?	Family living arrangements	0.00
Occupation: Packaging Worker	Occupation	0.00
Is your daily food intake in the past year higher than the reference amount? (reference amount:1/2 slice of cod or karei (40g))	Eating habits	0.00
Occupation: general office worker	Occupation	0.00
Were there any days in the week before your pregnancy when you walked continuously for more than 10 minutes?	Physical activity	0.00
Occupation: clerical worker	Occupation	0.00
During this pregnancy, did you handle organic solvent at work at least once a month?	Working condition	0.00
How often do you consume chinese cabbage?	Eating habits	0.00
Passive smoking up to Junior high school hours per day	Smoking habits	0.00
Is your daily food intake in the past year higher than the reference amount? (reference amount:1 small plate of pickles (cucumbers) 30 g))	Eating habits	0.00
Disease History: dysmenorrhea	Disease history	0.00

Occupation: sales-related position	Occupation	0.00
Are you living with your siblings?	Family living arrangements	0.00
Is your daily food intake in the past year higher than the reference amount? (reference amount: 1/3 potato, 50g)	Eating habits	0.00
Is your daily food intake in the past year higher than the reference amount? (reference amount:1 small plate of pickled vegetables (nozawana or takana(30 g))	Eating habits	0.00
How often do you consume cod roe?	Eating habits	0.00
How often do you consume sweet potato?	Eating habits	0.00
How often do you consume cod roe?	Eating habits	0.00
How often do you consume grilled fish?	Eating habits	0.00
Is your daily food intake in the past year higher than the reference amount? (reference amount: 80 g per horse mackerel or sardine)	Eating habits	0.00
Is your daily food intake in the past year higher than the reference amount? (reference amount: 2 heads of spinach, 50g)	Eating habits	0.00
Have your eating habits changed significantly in the past 5 years?	Eating habits	0.00
Is your daily food intake in the past year higher than the reference amount? (reference amount:1/4 canned tuna 20g)	Eating habits	0.00
How often do you consume horse mackerel and sardine?	Eating habits	0.00
Is your daily food intake in the past year higher than the reference amount? (reference amount: 1/2 of a peach, 65g)	Eating habits	0.00
Duration of passive smoking by junior high school students (years)	Smoking habits	0.00
Did you use any medications or supplements between 1 year before and when you found out you were pregnant?	Medication	0.00
How often do you consume pacific saury and mackerel?	Eating habits	0.00
Is your daily food intake in the past year higher than the reference amount? (reference amount: 200cc of milk, 1 bottle)	Eating habits	0.00
Diagnosis received during past pregnancy: threatened preterm labor	Disease history	0.00
Is your daily food intake in the past year higher than the reference amount? (reference amount: 1 pumpkin, cut into 4~5cm cubes, 40g)	Eating habits	0.00
Is your daily food intake in the past year higher than the reference amount? (reference amount:1/3 of an octopus leg (50g))	Eating habits	0.00
Occupation: product inspection (metal products)	Occupation	0.00
How sleepy are you during the day?	Sleeping condition	0.00
How often do you consume eggplant?	Eating habits	0.00
Is your daily food intake in the past year higher than the reference amount? (reference amount: 1/2 tbsp mayonnaise, 7g)	Eating habits	0.00
How many days a week do you work?	Working condition	0.00
Is your daily food intake in the past year higher than the reference amount? (reference amount:4 slices of Buri/Hamachi sashimi(60g))	Eating habits	0.00
Is your daily food intake in the past year higher than the reference amount? (reference amount: 1/4 of enoki mushrooms, 20g)	Eating habits	0.00
How long does it take to fall asleep?	Sleeping condition	0.00
Is your daily food intake in the past year higher than the reference amount? (reference amount:1/4 tomato (50g))	Eating habits	0.00

Passive smoking in the year before pregnancy hours per day	Smoking habits	0.00
Is your daily food intake in the past year higher than the reference amount? (reference amount:1 slice 70g salted cod, salted Hokke, or salted salmon)	Eating habits	0.00
Is your daily food intake in the past year higher than the reference amount? (reference amount: 1/8 of a pineapple, 130g)	Eating habits	0.00
