# Supplementary material for: Early prediction of hypertensive disorders of pregnancy toward preventive early intervention
Source: AJOG Glob Rep. 2024 Jul 27;4(4):100383. doi: 10.1016/j.xagr.2024.100383 (PMC11550347; doi:10.1016/j.xagr.2024.100383)
Supplement: Supplementary file 7 [file mmc7.pdf]

Supplementary Table 5 : The F1-scores for both early- and full-term prediction models

HDP-nonHDP model

Datasets	Type of prediction model	Feature selection	Machine learning models				
			Logistic	RF	SVM	DNN	XGBOOST
Laboratory test data collected in the early stage of pregnancy	Early prediction model	HSIC LASSO	0.81	0.84	0.85	0.79	0.85
		RFE	0.81	0.84	0.86	0.79	0.85
Questionnaires completed in the early stage of pregnancy	Early prediction model	HSIC LASSO	0.87	0.87	0.90	0.87	0.89
		RFE	0.88	0.86	0.93	0.91	0.89
Questionnaires completed in the late stage of pregnancy	Full-term prediction model	HSIC LASSO	0.84	0.84	0.88	0.84	0.86
		RFE	0.87	0.85	0.91	0.9	0.88
Medical record of first visit interview	Full-term prediction model	HSIC LASSO	0.77	0.84	0.84	0.83	0.85
		RFE	0.76	0.80	0.81	0.78	0.80
Prenatal checkup data 2	Full-term prediction model	HSIC LASSO	0.86	0.85	0.90	0.87	0.88
		RFE	0.89	0.87	0.93	0.94	0.90
Prenatal checkup data 3	Full-term prediction model	HSIC LASSO	0.86	0.87	0.89	0.88	0.89
		RFE	0.90	0.87	0.93	0.90	0.89
Concatenated dataset 1	Full-term prediction model	HSIC LASSO	0.87	0.88	0.90	0.89	0.89
		RFE	0.92	0.88	0.94	0.93	0.92
Concatenated dataset 2	Full-term prediction model	HSIC LASSO	0.88	0.88	0.91	0.90	0.89
		RFE	0.92	0.88	0.94	0.94	0.91
Concatenated dataset 3	Full-term prediction model	HSIC LASSO	0.89	0.89	0.90	0.91	0.92
		RFE	0.92	0.89	0.94	0.93	0.92

GH-(SPE/PE) model

Datasets	Type of prediction model	Feature selection	Machine learning models				
			Logistic	RF	SVM	DNN	XGBOOST
Laboratory test data collected in the early stage of pregnancy	Early prediction model	HSIC LASSO	0.56	0.57	0.56	0.52	0.58
		RFE	0.58	0.53	0.58	0.51	0.53
Questionnaires completed in the early stage of pregnancy	Early prediction model	HSIC LASSO	0.53	0.52	0.52	0.51	0.55
		RFE	0.59	0.50	0.59	0.52	0.53
Questionnaires completed in the late stage of pregnancy	Full-term prediction model	HSIC LASSO	0.54	0.52	0.53	0.52	0.54
		RFE	0.55	0.52	0.54	0.51	0.53
Medical record of first visit interview	Full-term prediction model	HSIC LASSO	0.52	0.48	0.50	0.50	0.48
		RFE	0.52	0.52	0.53	0.50	0.53
Prenatal checkup data 2	Full-term prediction model	HSIC LASSO	0.62	0.65	0.63	0.54	0.64
		RFE	0.64	0.64	0.64	0.54	0.63
Prenatal checkup data 3	Full-term prediction model	HSIC LASSO	0.61	0.62	0.61	0.54	0.63
		RFE	0.63	0.63	0.62	0.52	0.61
Concatenated dataset 1	Full-term prediction model	HSIC LASSO	0.60	0.61	0.60	0.54	0.62
		RFE	0.67	0.60	0.67	0.51	0.62
Concatenated dataset 2	Full-term prediction model	HSIC LASSO	0.60	0.64	0.61	0.57	0.64
		RFE	0.63	0.61	0.62	0.52	0.63
Concatenated dataset 3	Full-term prediction model	HSIC LASSO	0.60	0.63	0.62	0.56	0.64
		RFE	0.62	0.61	0.62	0.53	0.62

SPE-PE model

Datasets	Type of prediction model	Feature selection	Machine learning models				
			Logistic	RF	SVM	DNN	XGBOOST
Laboratory test data collected in the early stage of pregnancy	Early prediction model	HSIC LASSO	0.68	0.67	0.70	0.53	0.67
		RFE	0.70	0.70	0.70	0.59	0.67
Questionnaires completed in the early stage of pregnancy	Early prediction model	HSIC LASSO	0.65	0.69	0.68	0.56	0.69
		RFE	0.71	0.67	0.75	0.62	0.70
Questionnaires completed in the late stage of pregnancy	Full-term prediction model	HSIC LASSO	0.62	0.68	0.69	0.55	0.68
		RFE	0.69	0.64	0.71	0.61	0.63
Medical record of first visit interview	Full-term prediction model	HSIC LASSO	0.65	0.65	0.67	0.54	0.64
		RFE	0.71	0.73	0.71	0.57	0.72
Prenatal checkup data 2	Full-term prediction model	HSIC LASSO	0.74	0.71	0.72	0.54	0.72
		RFE	0.78	0.72	0.82	0.66	0.69
Prenatal checkup data 3	Full-term prediction model	HSIC LASSO	0.72	0.71	0.72	0.61	0.71
		RFE	0.78	0.73	0.78	0.53	0.75
Concatenated dataset 1	Full-term prediction model	HSIC LASSO	0.74	0.73	0.73	0.67	0.74
		RFE	0.76	0.70	0.75	0.61	0.71
Concatenated dataset 2	Full-term prediction model	HSIC LASSO	0.71	0.74	0.72	0.66	0.74
		RFE	0.78	0.70	0.81	0.79	0.72
Concatenated dataset 3	Full-term prediction model	HSIC LASSO	0.71	0.73	0.72	0.62	0.74
		RFE	0.77	0.73	0.76	0.54	0.75
