# Supplementary material for: Early prediction of hypertensive disorders of pregnancy toward preventive early intervention
Source: AJOG Glob Rep. 2024 Jul 27;4(4):100383. doi: 10.1016/j.xagr.2024.100383 (PMC11550347; doi:10.1016/j.xagr.2024.100383)
Supplement: Supplementary file 8 [file mmc8.pdf]

Supplementary Table 6 : The performance of the positive/negative controls

	LR	RF	SVM	DNN	XGBOOST
Positive Control	1.00	1.00	1.00	1.00	1.00
Negative Control	0.50	0.50	0.50	0.51	0.50
