# Supplementary material for: Early prediction of hypertensive disorders of pregnancy toward preventive early intervention
Source: AJOG Glob Rep. 2024 Jul 27;4(4):100383. doi: 10.1016/j.xagr.2024.100383 (PMC11550347; doi:10.1016/j.xagr.2024.100383)
Supplement: Supplementary file 9 [file mmc9.pdf]

Supplementary Table 7 : Number of selected features by feature selection
HSIC LASSO

Datasets	Conversion method of distributon	Number of selected variables (*n* , %)		
		Prediction models		
		HDP-nonHDP	GH-(SPE/PE)	SPE-PE
Laboratory test data collected in the early stage of pregnancy	Box-Cox transformation	14 (51.85)	14 (51.85)	14 (51.85)
	Box-Cox transformation plus scaling	16 (61.54)	16 (61.54)	16 (61.54)
	Scaling	16 (61.54)	16 (61.54)	16 (61.54)
Questionnaires completed in the early stage of pregnancy	Box-Cox transformation	108 (21.82)	150 (30.30)	120 (24.24)
	Box-Cox transformation plus scaling	165 (33.40)	260 (52.63)	189 (38.26)
	Scaling	181 (36.57)	226 (45.66)	189 (38.18)
Questionnaires completed in the late stage of pregnancy	Box-Cox transformation	112 (19.82)	179 (31.68)	122 (21.59)
	Box-Cox transformation plus scaling	222 (39.29)	272 (48.14)	202 (35.75)
	Scaling	226 (40.00)	258 (45.66)	192 (33.98)
Medical record of first visit interview	Box-Cox transformation	6 (4.62)	8 (6.15)	8 (6.15)
	Box-Cox transformation plus scaling	55 (42.31)	Not comvergence	Not comvergence
	Scaling	55 (42.31)	Not comvergence	Not comvergence
Prenatal checkup data 2	Box-Cox transformation	104 (10.67)	117 (12.00)	1 (0.10)
	Box-Cox transformation plus scaling	113 (11.60)	165 (16.94)	121 (12.42)
	Scaling	35 (3.62)	153 (15.82)	120 (12.41)
Prenatal checkup data 3	Box-Cox transformation	102 (9.03)	116 (10.27)	78 (6.90)
	Box-Cox transformation plus scaling	94 (8.31)	159 (14.06)	100 (8.84)
	Scaling	53 (4.69)	159 (14.06)	113 (9.99)
Concatenated dataset 1	Box-Cox transformation	127 (7.54)	205 (12.17)	136 (8.08)
	Box-Cox transformation plus scaling	305 (18.11)	311 (18.47)	265 (15.74)
	Scaling	307 (18.23)	300 (17.81)	200 (11.88)
Concatenated dataset 2	Box-Cox transformation	135 (6.28)	223 (10.38)	134 (6.24)
	Box-Cox transformation plus scaling	264 (12.28)	426 (19.81)	263 (12.23)
	Scaling	255 (11.86)	384 (17.86)	219 (10.19)
Concatenated dataset 3	Box-Cox transformation	131 (5.58)	232 (9.89)	116 (4.94)
	Box-Cox transformation plus scaling	256 (10.90)	318 (13.54)	237 (10.09)
	Scaling	245 (10.44)	318 (13.55)	249 (10.61)

RFE

Datasets	Conversion method of distributon	Number of selected variables (*n* , %)		
		Prediction models		
		HDP-nonHDP	GH-SPE/PE	SPE-PE
Laboratory test data collected in the early stage of pregnancy	Box-Cox transformation	23 (85.19)	1 (3.70)	12 (44.44)
	Box-Cox transformation plus scaling	25 (96.15)	1 (3.85)	14 (53.85)
	Scaling	24 (92.31)	1 (3.85)	22 (84.62)
Questionnaires completed in the early stage of pregnancy	Box-Cox transformation	252 (50.91)	38 (7.68)	24 (4.85)
	Box-Cox transformation plus scaling	268 (54.25)	2 (0.40)	223 (45.14)
	Scaling	372 (75.15)	4 (0.81)	350 (70.71)
Questionnaires completed in the late stage of pregnancy	Box-Cox transformation	141 (24.96)	396 (70.09)	168 (29.73)
	Box-Cox transformation plus scaling	206 (36.46)	1 (0.18)	3 (0.53)
	Scaling	309 (54.69)	3 (0.53)	233 (41.24)
Medical record of first visit interview	Box-Cox transformation	18 (13.85)	1 (0.77)	23 (17.69)
	Box-Cox transformation plus scaling	7 (5.38)	54 (41.54)	1 (0.77)
	Scaling	4 (3.08)	1 (0.77)	4 (3.08)
Prenatal checkup data 2	Box-Cox transformation	149 (15.28)	56 (5.74)	50 (5.13)
	Box-Cox transformation plus scaling	226 (23.20)	227 (23.31)	135 (13.86)
	Scaling	212 (21.92)	159 (16.44)	161 (16.65)
Prenatal checkup data 3	Box-Cox transformation	63 (5.58)	43 (3.81)	6 (0.53)
	Box-Cox transformation plus scaling	604 (53.40)	8 (0.71)	73 (6.45)
	Scaling	386 (34.13)	9 (0.80)	76 (6.72)
Concatenated dataset 1	Box-Cox transformation	986 (58.55)	1 (0.06)	828 (49.17)
	Box-Cox transformation plus scaling	141 (8.37)	118 (7.01)	23 (1.37)
	Scaling	153 (9.09)	1 (0.06)	38 (2.26)
Concatenated dataset 2	Box-Cox transformation	147 (6.84)	1 (0.05)	1003 (46.69)
	Box-Cox transformation plus scaling	254 (11.81)	16 (0.74)	28 (1.30)
	Scaling	136 (6.33)	17 (0.79)	24 (1.12)
Concatenated dataset 3	Box-Cox transformation	66 (2.81)	5 (0.21)	2 (0.09)
	Box-Cox transformation plus scaling	191 (8.13)	7 (0.30)	41 (1.75)
	Scaling	318 (13.55)	4 (0.17)	340 (14.49)
