# Supplementary material for: Early prediction of hypertensive disorders of pregnancy toward preventive early intervention
Source: AJOG Glob Rep. 2024 Jul 27;4(4):100383. doi: 10.1016/j.xagr.2024.100383 (PMC11550347; doi:10.1016/j.xagr.2024.100383)
Supplement: Supplementary file 10 [file mmc10.pdf]

Supplementary Table 8 : The performance of the bagging models

Datasets	HDP-nonHDP	GH-(SPE/PE)	SPE-PE
Laboratory test data collected in the early stage of pregnancy	0.58	0.57	0.57
Questionnaires completed in the early stage of pregnancy	0.60	0.52	0.59
Questionnaires completed in the late stage of pregnancy	0.55	0.53	0.56
Medical record of first visit interview	0.59	0.52	0.59
Prenatal checkup data 2	0.77	0.62	0.65
Prenatal checkup data 3	0.82	0.63	0.67
Concatenated dataset 1	0.76	0.62	0.64
Concatenated dataset 2	0.77	0.64	0.66
Concatenated dataset 3	0.82	0.63	0.67
